# Hypnotics as induction agents for general anesthesia in cesarean section patients: updated systematic review and meta-analysis of randomized controlled trials

**DOI:** 10.1007/s00540-025-03524-8

**Published:** 2025-06-21

**Authors:** Sohieb Hedawy, Eman E. Labeeb, Shahed Aldalahmeh, Ahmed Elswaf, Ghadeer M. AlManaseer, Mohamed Ashraf Shehab, Ahmed Menshawy

**Affiliations:** 1 Faculty of Medicine, Al-Azhar Assiut University, Assiut, Egypt; 2 Faculty of Medicine, Al-Azhar Cairo University, Cairo, Egypt; 3 Faculty of Medicine, Jordan University of Science and Technology, Al-Ramtha, Jordan; 4 University of Jordan Hospital, Amman, Jordan; 5 Faculty of Medicine, Menoufia University, Menoufia, Egypt; 6 Obs/Gyn Department, Faculty of Medicine, Al Hussien University Hospital, Al Azhar University, Cairo, Egypt

**Keywords:** Cesarean section, General anesthesia, Thiopental, Propofol, And ketamine

## Abstract

**Background and purpose:**

General anesthesia is indicated in emergencies, contraindications, or patient requests. The induction agent to use is an important factor in general anesthesia. We aim to provide an updated systematic review and meta-analysis to compare propofol, ketamine, and thiopental sodium in terms of efficacy and safety profiles in women undergoing cesarean sections under general anesthesia.

**Methods:**

We conducted this systematic review and meta-analysis according to PRISMA guidelines. We searched the following databases (PubMed, Scopus, Cochran Library, and Web of Science) up to 18-8-2024. We used a term for cesarian section, thiopental, ketamine, and propofol.

**Results:**

Thirty-six randomized controlled trials met our criteria and were included in our analysis with a total of 1945 patients. In (Thiopentone vs. Propofol) group, the use of thiopentone was associated with higher umbilical artery PH and longer recovery duration, although the certainty of evidence was low for both outcomes, while in the propofol group, the risk of having neonates with Apgar score less than 7 at one minute was higher, but the certainty of evidence was very low. In (Thiopentone vs. Ketamine) group, patients induced with thiopentone reported accidental awareness during general anesthesia (AAGA) more frequently and their neonates revealed higher umbilical vein PvO_2_. Also, Apgar score < 7 at 1 and 5 min were less frequent in the thiopentone group. The certainty of evidence for these outcomes was moderate. In (Propofol vs. Ketamine) group, there were no differences among reported outcomes, and the certainty of evidence was low to very low**.**

**Conclusion:**

Our findings suggest that propofol and thiopentone appear to be clinically comparable. Ketamine was favored over thiopentone for its lower risk of AAGA, while thiopentone was associated with a lower risk of Apgar scores < 7 at 1 and 5 min. Additional well-designed trials are needed to support our conclusions more firmly.

**Supplementary Information:**

The online version contains supplementary material available at 10.1007/s00540-025-03524-8.

## Introduction

In recent years, the rate of Cesarean section (CS) has increased globally [1, 2]. According to the World Health Organization (WHO), CS accounts for 21% of all childbirths and is projected to reach 29% in 2030 [3]. Regional anesthesia is currently the most common method used in CS; however, General anesthesia is still indicated in emergencies, contraindications, or patient requests [4, 5]. Which induction agent to use is an important factor in general anesthesia.

Ideal induction agents should rapidly anesthetize the mother, and maintain constant maternal hemodynamics, with minimal neonatal depression [6, 7]. Thiopental sodium was the first induction agent introduced by Lundy J. S. in 1935 as an induction agent for mothers undergoing elective or emergency cesarean section under general anesthesia [8]. Despite its several downsides; including low maternal arterial blood pressure, crossing the placenta, and causing fetal depression, thiopental sodium was still widely used in obstetric anesthesia and considered the standard to which new anesthetic agents are compared [7, 9]. In 2011, the use of thiopental declined as the companies producing it in France and the United States stopped manufacturing it for political and economic reasons, which led to an increase in the Consumption of other anesthetic agents in obstetrics [10].

Several anesthetic agents have been recommended as alternatives for thiopental, including propofol and ketamine [11–15]. However, these agents also cross the placenta and thus can depress the fetal nervous system [6, 16–20]. several studies have compared propofol, ketamine, and thiopental leading to controversies regarding the ideal drug to use [12, 14, 20–22]. A previous systematic review was conducted in 2018 to investigate the efficacy and safety profiles of a variety of hypnotic agents in women undergoing CS under general anesthesia [23]. However, several studies have been carried out since then showing conflicting results [24–26].

Therefore, we aim to provide an updated systematic review and meta-analysis to compare propofol, ketamine, and thiopental sodium in terms of efficacy and safety profiles, in women undergoing cesarean section under general anesthesia.

## Methods

This systematic review and meta-analysis was performed according to preferred reporting items for systematic review and meta-analysis (PRISMA), agreed with the guidelines in Cochrane's Handbook of Systematic Reviews of Interventions and registered in PROSPERO(CRD42024622419).

### Search strategy and selection criteria

One investigator searched PubMed, Cochrane Library, Web of Science, and Scopus databases up to 18 August 2024. The search strategy included the Medical Subject Headings (MeSH) and text words for the following terms: “cesarean section” and “propofol,” “thiopentone, “, “ketamine,” and “etomidate” (Supplementary table). Although etomidate was included in our search strategy, no eligible studies with sufficient data for quantitative analysis were identified, and it was therefore not included in the final meta-analysis.

The inclusion criteria for studies published in international and peer-reviewed journals were as follows: (a) randomized controlled trials of participants undergoing cesarean section with general anesthesia induced by one of these agents: Thiopentone, Propofol, and Ketamine; (b) at least one of these primary outcomes have to be reported: Apgar score, arterial or venous umbilical blood gasses (pH, PO_2_, PCO_2_, HCO_3_ and Base excess) (c) other secondary outcomes were included such as Accidental awareness during general anesthesia, recovery duration, and the change in SBP, DBP and heart rate. There are no language, age, or country restrictions.

The exclusion criteria were as follows: (a) not a randomized clinical trial; (b) the mode of anesthesia is not general anesthesia; (c) studies with a combination of any of those induction agents; (d) unavailable full text.

The duplicates were then removed using Endnote software. Two independent reviewers screened the articles using Excel in two steps: titles and abstracts, followed by full texts, and those that matched our criteria were included. A third independent reviewer was consulted to resolve any conflict.

### Data extraction and quality assessment

Two independent investigators used an Excel spreadsheet to extract the following information from eligible studies: the first author’s name, year of publication, country, aim of the study, comparison agents, dosage of induction agents (mg/kg), number of patients in each group, primary endpoints, maintenance of anesthesia drugs, and the conclusion. We extracted maternal age, gestational age, as well as neonatal and maternal weight as baseline characteristics. Outcomes extracted were Apgar score, arterial or venous umbilical blood gasses or maternal blood gases (pH, PO_2_, PCO_2_, and Base excess), SPO_2_ and HCO_3_ in the uterine artery and vein, dreams, recall, anesthesia duration (min), recovery duration(sec), change in SBP, DBP, and HR before and after induction of anesthesia and uterine incision to delivery time (sec.). Recovery duration was defined as the time (in seconds) from the end of anesthesia administration until the patient met predefined recovery criteria, such as the ability to open eyes on command and follow simple instructions. A third investigator cross-checked to resolve any errors.

Two investigators assessed the quality of the studies according to the Cochrane risk of bias (ROB2) criteria for randomized controlled studies (RCTs). RCT studies were graded as "low risk”, "some concerns” or “high risk" according to the following five domains: randomization process, deviations from the intended interventions, missing outcome data, measurement of the outcome, and selection of the reported result.

### Statistical analysis

We used Review Manager Version 5.4.1, the random-effects model in performing meta-analysis. We evaluated the dichotomous data using risk ratios with 95% confidence intervals. If the data exists as median and IQR, we convert it to mean and SD. Heterogeneity is reported using I^2^ and Chi^2^ tests; I^2^ > 50% indicates significant heterogeneity. When there was significant heterogeneity between the two included studies, we performed manual sensitivity analysis by excluding one study from each analysis, and finally, we performed a leave-one-out meta-analysis to solve the heterogeneity.

Also, We conducted trial sequential analysis (TSA) to assess quantitatively the need for further trials with a larger sample size. We used Trial Sequential Analysis (TSA) [Computer program] (Version 0.9.5.10 Beta. The Copenhagen Trial Unit, Centre for Clinical Intervention Research, The Capital Region, Copenhagen University Hospital – Rigshospitalet, 2021). The TSA followed standardized assumptions, including a Type I error of 5%, 80% power, O'Brien-Fleming model in Alpha Spending boundaries, variance-based heterogeneity correction, and empirically determined mean difference and variance.

### Certainty assessment

The certainty of evidence was assessed using the Grading of Recommendations Assessment, Development and Evaluation (GRADE) approach. Two independent reviewers conducted the assessment using GRADEpro GDT: GRADEpro Guideline Development Tool [Software]. McMaster University and Evidence Prime, 2025. Available from gradepro.org. Disagreements between reviewers were resolved by discussion or consultation with a third reviewer. The certainty of evidence for each primary outcome was classified as high, moderate, low, or very low based on the following GRADE domains: risk of bias, inconsistency, indirectness, imprecision, and publication bias. Summary-of-findings tables were generated and are provided as supplementary data.

## Results

### Study selection

**Fig. 1:**
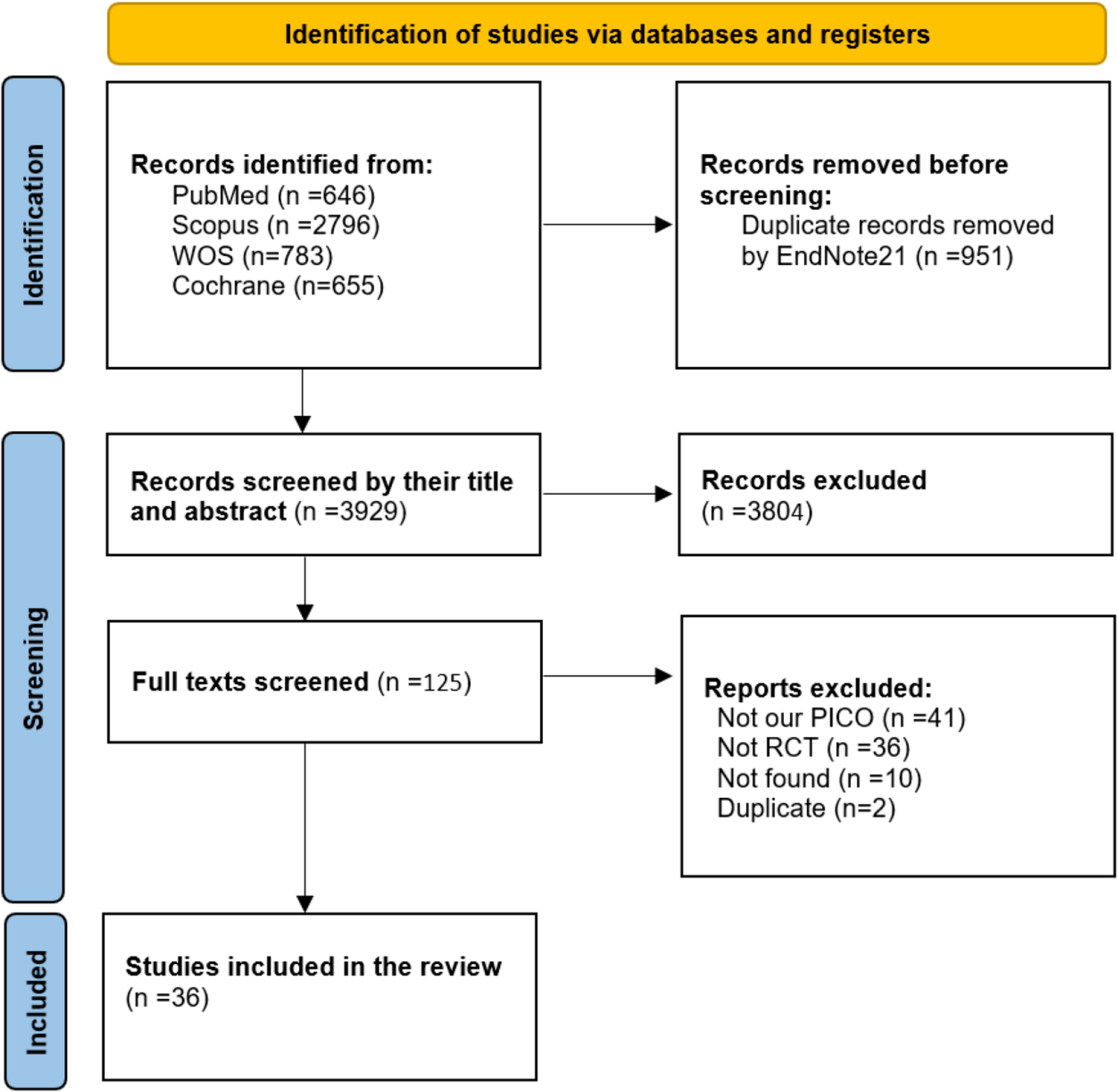
The PRISMA flow diagram of studies screening and selection

The electronic search yielded 4880 records of which 951 were duplicates. A total of 3804 were excluded by title and abstract screening. The remaining 125 records were further evaluated by full-text screening and only 36 clinical trials [6, 7, 11–14, 19–22, 25, 27–51] met our inclusion criteria (Fig. 1).

### Characteristics of included studies

A total of 1945 patients were included in our studies,1339 patients in (thiopentone vs. propofol) group; 502 patients in (thiopentone vs. ketamine) group; and only 170 patients in (propofol vs. ketamine) group. Various agents were used to maintain anesthesia including propofol in 5 studies; The maternal age and weight, gestational age, and neonatal weight are reported in Table 1. Our main primary outcomes were the Apgar score and umbilical blood gases (pH, PO_2_, PCO_2_, BE, SPO_2_, and HCO_3_). Baraka et al., 1989 used two maintenance regimens, 50%NO + 0.5%Halothane in Oxygen for group 1&3 and 50%NO + 1%Halothane in 100% Oxygen for group 2&4. For analysis purposes, we presented them as Baraka 1 for the first regimen and Baraka 2 for the second regimen. Groups in which anesthesia was induced with a combination of agents were not included in our analysis. Trials with insufficient data were excluded from the analysis.

**Table 1:** Summary of included studies and characteristics of their population

Study	Country	Treatment groups(n)	Dos of induction (mg/kg)	Primary outcomes	Maintenance of anesthesia drugs	Aim of the study	Conclusion of the most important results	Maternal age (years) Mean ± SD	Maternal weight (kg) Mean ± SD	Gestational age(weeks) Mean ± SD	Neonatal weight(g) Mean ± SD
Abboud et al., 1995 [27]	Denmark	1)Propofol(37) 2)Thiamylal(37)	1) 1.5:2 2) 3:4	BP, Apgar score and UBGs	1)propofol/N2O 2) isoflurane/N2O	To compare both drug groups regards BP, Apgar score, and umbilical blood gases	Neonatal status as ascertained by APGAR scores. Cord acid–base status and the neurological and adaptive capacity scores were equally good in both groups. It is concluded that propofol used for induction and maintenance of anesthesia is a safe alternative for Thiamylal/Isoflurane for CS patients and is associated with less hypertensive response during laryngoscopy and intubation	30.2 ± 1 29.5 ± 1	78.7 ± 2.1 78.2 ± 2.1	39.2 ± 0.3 39.2 ± 0.3	3821 ± 112 3949 ± 77
Baraka et al., 1989*1 [22]	Lebanon	1)Thiopentone + Maintenance 1 (10) 2)Thiopentone + Maintenance 2(10) 3)Ketamine + Maintenance 1 (10) 4)Ketamine + Maintenance 2 (10)	1) 4 2) 4 3) 1.5 4) 1.5	Awareness, Apgar scores, and UBGs	1):(NO + 0.5%Halothan in Oxygen) 2):(NO + 1%Halothan in Oxygen) 3):(NO + 0.5%Halothan in Oxygen) 4):(NO + 1%Halothan in Oxygen)	To examine the incidence of awareness in patients undergoing Caesarean section, compared with other techniques of general anesthesia	Awareness was significantly greater after induction with thiopentone than after ketamine. There were no significant differences in Apgar scores or umbilical vein blood-gas values in newborns	31 ± 4.9	71 ± 8.3	NA	NA
Baraka et al., 1997 [28]	Lebanon	1)Thiopentone(20) 2)Ketamine(20)	1) 4 2) 1.5	Apgar score and recovery duration	1)N2O:O2 mixture (1:1) + 0.5% halothane 2)100% oxygen only	To investigate the neuromuscular effects and conditions of tracheal intubation in ketamine-rocuronium versus thiopental-rocuronium in 40 patients undergoing elective cesarean section	Time to 50% NMB & The onset time were not significantly different between the two groups. Tracheal intubation at 50% NMB was easily performed in all patients in the Ketamine-rocuronium group but difficult in 75% of the thiopental-rocuronium group	31 ± 6 31 ± 5	76 ± 13 72 ± 10	NA NA	NA NA
Bernstein et al., 1985 [29]	Sweden	1)Thiopentone(12) 2)Ketamine(16)	1) 4 2) 1	The ID-times, UBGs, and Apgar scores	50% 02 + 50% N20	To investigate the influence of thiopentone versus ketamine on the newborn and the correlation between fetal oxygenation and induction-delivery time in elective cesarean section	The ID times were varied between 2 and 10 min. The PO_2_, acid–base values, and Apgar scores did not differ between the two groups. A significant negative correlation between the PO_2_ of the newborn at the moment of birth and the ID time was found in the thiopentone group	30 33	70.9 75.6	NA NA	3158 3276
Çakırtekin et al., 2015 [30]	Turkey	1)Thiopental(35) 2)propofol(35)	1) 5 2) 2	Awareness, APGAR scores, and UBGs (VAS) scores	sevoflurane + O2 + N2O	To compare the effects of propofol and thiopental on hemodynamics, awareness, and newborns in pregnant women under Going elective cesarean section	SBP, MAP, and DBP at the first 2 min after induction and heart rate (HR) at almost all time points were significantly higher in the thiopental group. No patient remembered the keyword spelled, while 4 patients reported dreaming during general anesthesia. The effects of propofol and thiopental sodium on 1 and 5-min APGAR scores, and cord blood gases were similar	28.43 ± 4.5 29.2 ± 5.71	81.23 ± 10.06 78.94 ± 11.32	38.97 ± 0.28 39.03 ± 0.32	NA NA
Capogna et al., 1991 [19]	Italy	1)Thiopentone(28) 2)Propofol(28)	1) 4.84 ± 1.24 2) 2.36 ± 0.57	Maternal hemodynamics, Awareness, Apgar score, and UBGs	NO + oxygen + isoflurane	To compare thiopentone with propofol for induction of anesthesia in patients scheduled for an elective cesarean section concerning fetal blood gases, APGAR score, and maternal hemodynamics	In the thiopentone group, the arterial pressure rose following tracheal intubation, while in the propofol group, there was a significant tendency to hypotension immediately following the induction of anesthesia. There were differences in EEG between the groups. Neonates in the propofol group had non-significant lower Apgar scores 1 min after birth	30.96 ± 4.31 30.18 ± 7.53	74.9 ± 11.7 71.1 ± 17.4	39.39 ± 1.14 39.52 ± 1.58	3282.73 ± 580.3 3230.91 ± 418.23
Celleno et al., 1993 [31]	Italy	1)Thiopental(30) 2)Propofol(30)	1) 5 2) 2.4	Maternal hemodynamics, Recovery time, and Apgar score	N2O + oxygen + isoflurane	To evaluate maternal hemodynamics, depth of anesthesia, and neonatal APGAR scores after induction of anesthesia with thiopental, propofol, or midazolam for elective cesarean section	SBP and HR rose after intubation and skin incision in thiopental and midazolam groups, while in the propofol group, there was significant hypotension following induction. EEG patterns showed a light depth of anesthesia in midazolam and propofol groups between induction and delivery. In the midazolam and propofol groups, the neonates had lower APGAR and neurobehavioral scores than the thiopental group. Umbilical artery-to-vein ratios were above 1 in the midazolam and propofol groups	31.2 ± 8.6 31.6 ± 4.7	76.4 ± 12.3 69.4 ± 6.2	38.3 ± 1.4 37.9 ± 1.8	3425.5 ± 377.9 3412.3 ± 368.7
Celleno et al., 1989 [20]	Italy	1)Thiopentone(20) 2)Propofol(20)	1) 5 2) 2.8	ENNS scores and Apgar score	50% nitrous oxide in oxygen	To examine the neurobehavioral effects of propofol on neonates when given as an induction agent for Caesarean section	Infants in the propofol group had lower Apgar scores at 1 and 5 min	30.9 ± 5.5 30.9 ± 4.5	69.6 ± 6.2 69.7 ± 9.1	40.1 ± 0.5 39.6 ± 0.4	3412.5 ± 368.7 3326 ± 457.6
Giatini et al., 1995 [50]	Israel	1)Thiopentone(25) 2)Ketamine(25)	1) 4 2) 1	Awareness and Apgar scores	50%nitrous oxide and 50% oxygen	To examine the relationship between the EEG and the occurrence of awareness	The incidence of responsiveness to verbal commands was lower in the ketamine group. The results suggest that SEF values of < 8.6 Hz were sufficient to avoid responsiveness to verbal commands	26.2 ± 5 25.5 ± 5	72.8 ± 7 72 ± 9	NA NA	NA NA
Gin et al., 1990 [13]	China	1)Propofol(20) 2)Thiopentone(20)	1) 2 2) 4	Maternal hemodynamics, Apgar score, and Awareness	enflurane + N2O + O2	To compare Propofol versus Thiopentone for Induction of Caesarean Section as regards hemodynamics	Induction with propofol causes less variation in arterial pressure than thiopentone. Hypotension is probably prevented by the coincident stimulus of rapid sequence induction. Neonatal Apgar scores were similar between the two groups	30 ± 5.4 28.7 ± 4.5	62.4 ± 8.3 62.7 ± 8.3	39.2 ± 1 38.6 ± 1	3180 ± 440 3060 ± 320
Gin et al., 1993 [32]	China	1)Propofol(30) 2)Thiopentone(32)	1) 2 2) 4	Maternal hemodynamics and neonatal and maternal blood gases	isoflurane + N2O + O2	To compare Propofol versus Thiopentone for Induction of Caesarean Section concerning hemodynamics, and maternal and neonatal blood gases	The increase from baseline values in MAP after intubation was greater in the thiopentone group compared with the propofol group. Neonatal Apgar scores, neurobehavioral testing, blood gases, and oxygen content analysis were similar between groups	30.9 30.6	63 ± 8.8 63.7 ± 8.5	38.4 ± 1.1 38.6 ± 0.9	3100 ± 420 3230 ± 390
Hadav et al., 2019 [25]	Iran	1)Thiopental(45) 2)Propofol(45)	1) 0.5 2) 2.5	IFT values, BIS scores, recall	N2O + O2 + sevoflurane	To compare Thiopental and Propofol as regards postoperative recall	No patient recalled the perioperative events during the follow-up period. In the propofol group, BIS scores were significantly lower than the thiopental group after induction of GA until discontinuation of volatile anesthetics. IFT values were significantly higher in the thiopental group in the time between induction to skin incision compared to the propofol group	30.4 ± 52.7 31.5 ± 11.1	80.8 ± 24.52 81.1 ± 11.72	NA NA	NA NA
Houlto et al., 1978*2 [33]	South Africa	1)Thiopentone(38) 2)ketamine(45)	1) 3.5 2) 1	UBGs	enflurane + N2O + O2	To compare Thiopentone, Methohexitone, and Ketamine concerning maternal and fetal blood gases	The Thiopentone group was superior concerning fetal oxygenation, but blood gas/acid–base status was otherwise comparable among the different agents	25.8 ± 0.7 25.1 ± 0.6	70.4 ± 1.6 68.7 ± 1.2	NA NA	NA NA
Hwang et al., 2001 [34]	Korea	1)Thiopentone(34) 2)Propofol(34)	1) 5 2) 2	Maternal hemodynamics, Apgar score, and UBGs	Propofol + N2O	To compare thiopental sodium with propofol under propofol-N2O anesthesia as regards fetal and maternal blood gases and APGAR score	No significant difference in BP, HR, and Apgar scores between groups.PvO_2_ and oxygen saturation of the umbilical vein in the propofol group were higher than in the thiopentone group. PvCO_2_ and pH did not differ between groups	30.3 ± 3.7 30.2 ± 3.5	69.8 ± 10.2 69.2 ± 9.1	38.5 ± 0.9 38.7 ± 0.8	NA NA
Jeong et al., 1994 [35]	Korea	1)Thiopental(20) 2)ketamine(20) 3)Propofol(20)	1) 4 2) 1 3) 2.5	Maternal hemodynamics, Apgar score, and UBGs	enflurane + N2O + O2	to compare thiopental, ketamine, and propofol as induction agents in terms of Apgar score, maternal cardiovascular dynamics, and umbilical blood gas	No statically significant difference in the Apgar score between newborns among the three groups. The Thiopental group was higher regarding the pH of the umbilical artery and venous blood but with no statistically significant difference	27.7 ± 2.3 27.8 ± 2.9 27.8 ± 72	68.2 ± 5.4 66.8 ± 4.9 66.4 ± 7	NA	NA
Kim et al., 1997 *3 [37]	Korea	1)Propofol(15) 2)ketamine(15)	1) 2 2) 1	Maternal hemodynamics, Apgar score, and UBGs	enflurane + N2O + O2	To compare propofol, ketamine, and a propofol-ketamine combination on maternal and fetal outcomes, focusing on their impact on maternal hemodynamics and Apgar scores	The two groups were similar regarding the hemodynamic responses of mothers and newborns	28.4 ± 3.2 28.2 ± 2.1	67.7 ± 7.2 67.8 ± 6.1	NA	NA
Kim et al., 1994 [36]	Korea	1)Thiopental(15) 2)Propofol(15)	1) 4 2) 2	Maternal hemodynamics, Apgar score, and UBGs	enflurane + N2O + O2	To compare thiopental sodium with propofol as induction agents in cesarean sections for maternal hemodynamics, APGAR score, and umbilical blood gases	The two groups were similar regarding the hemodynamic responses of mothers and newborns. The difference was not significant	27.8 ± 4.2 28 ± 3.7	66.9 ± 8.1 65.5 ± 9.7	39.23 ± 1.4 39 ± 1.21	3200 ± 360 3360 ± 410
Lee et al., 2007 [38]	Korea	1)Thiopentone(23) 2)Propofol(22)	1) 4 2) 2	Maternal hemodynamics, Apgar score, and UBGs	isoflurane + N2O + O2	To compare propofol and thiopental effects on the anesthetic adequacy of pregnant who received inhalation anesthetics using the bispectral index (BIS) in the early period in SC	In the propofol group, systolic and diastolic pressure were significantly lower than in the Thiopental group immediately after intubation. All the BIS values from 0 to 9 min after intubation were significantly lower in the Propofol group than in the Thiopental group. The Apgar scores and umbilical cord blood gas analysis were similar in both groups	30.2 ± 3.6 31.1 ± 3.3	70.9 ± 5.6 73.3 ± 6.9	38.6 ± 0.8 38.5 ± 0.7	NA
Lee et al., 1993 [39]	Korea	1)Thiopentone(20) 2)Propofol(20)	1) 4 2) 2.5	Maternal hemodynamics and Apgar score	N2O + O2	To Compare the effects of Thiopental sodium with endotracheal intubation and Propfol with Laryngeal musk on BP and HR	thiopental sodium induction and tracheal intubation increase BP more than propofol and laryngeal mask in patients with preeclampsia	29 ± 3.6 27 ± 2.8	67 ± 6.5 69 ± 5.7	NA	NA
Mercan et al., 2012 [40]	Saudi arabia	1)Thiopentone(42) 2)Propofol(40)	1) 5 2) 2.5	Maternal hemodynamics and Apgar score	Sevoflurane	To compare the effect of propofol and thiopental for cesarean section on maintaining adequate BIS levels till the delivery	Anesthesia induction with propofol in a dose of 2.5 maintains lower levels of heart rate, blood pressure, and BIS till delivery when compared with thiopental in a dose of 5	30.8 ± 5.1 32.6 ± 4.3	79 ± 10.5 83.8 ± 10.8	38.6 ± 0.8 37.5 ± 1.4	NA
Ko et al., 1993 [41]	korea	1)Thiopentone(20) 2)Propofol(20)	1) 4 2) 2	Maternal hemodynamics, Apgar score, and UBGs	enflurane + N2O propofol + N2O	To compare thiopental sodium with propofol as induction agents for cesarean sections in terms of maternal hemodynamics, APGAR score, and umbilical blood gases	Hemodynamics were similar between groups except for diastolic and mean arterial pressure, there were fewer changes in the propofol group compared to the thiopental. Propofol group was higher as regards 1 min APGAR score	30.5 ± 4.57 31.2 ± 4.29	69.5 ± 7.48 62.9 ± 5.21	NA	NA
Moore et al., 1989 [11]	Ireland	1)Thiopental 2.5% (21) 2)Propofol 1% (21)	1) 4.53 ± 0.68 2) 2.15 ± 0.26	UBGs and Apgar Score	isoflurane + N2O + O2	To compare propofol and thiopentone used as induction agents for general anesthesia for patients who have elective Caesarean section as regards umbilical blood gases and APGAR score	Apgar score and umbilical blood gases showed little difference between the two groups. Factors associated with uterine relaxation and bleeding were similar in the two groups	29.4 ± 5.8 32.3 ± 6.3	74.5 ± 13.1 78.7 ± 12.5	38.1 ± 0.9 38.2 ± 0.6	NA
Kee et al., 1977 [42]	China	1)Thiopental (20) 2)Ketamine (18)	1) 4 2) 1	UBG	isoflurane + N2O + O2	to compare postoperative pain and analgesic requirement in the first 24 h in patients receiving ketamine vs thiopental	No patients experienced recall or unpleasant dreams. Apgar scores were similar between groups. Median umbilical venous pH was higher and attributable to lower median umbilical venous PvCO_2_ in the ketamine group compared with the thiopental group	31.3 ± 3.99 31.8 ± 4	62.8 ± 7.58 65.33 ± 13.68	NA	NA
Shraei et al., 2014 [43]	Iran	1)Thiopental (54) 2)Propofol (54)	1) 5:6 2) 2:2.5	Apgar score	Isofluran + NO + O2	To investigate the effect of Propofol and thiopental on the 1st and 5th minutes Apgar in neonate, hemodynamic conditions in mother under general anesthesia	There was a significant difference between groups as regards maternal hemodynamics. Propofol can be more appropriate than thiopental for induction of anesthesia in cesarean section and does not affect the hemodynamic changes and newborn Apgar score	NA	NA	NA	NA
schultetus et al., 1986 *4 [14]	USA	1)Ketamine(12) 2)Thiopental (13)	1) 1 2) 4	Maternal hemodynamics, Apgar score, and UBGs	succinylcholine chloride	to assess the effects of anesthetic induction with ketamine, thiopental, or a combination of the two drugs on maternal intraoperative awareness, recall, dreams, hallucinations, dysphoria, cardiovascular stability, fetal blood gas tensions, and Apgar and neurobehavioral scores of newborns	The use of Ketamine for induction of CS markedly decreased intraoperative responsiveness with few side effects. The combination of ketamine and thiopental, both at low doses, appears to offer little advantage over thiopental only concerning intraoperative responsiveness and was quite inferior to ketamine alone	23.5 ± 5.3 23.5 ± 4.5	75.9 ± 17.6 78.9 ± 17	NA	NA
Siafaka et al., 1992 [21]	Greece	1)Thiopental (10) 2)Propofol (10)	1) 4.4 2) 2.3	Apgar score, Umbilical and maternal blood gas	isoflurane + N2O + O2	to compare thiopental and propofol as induction agents in terms of Apgar score, umbilical and maternal blood gases, mean arterial blood pressure, and heart rate	From our study, we conclude that propofol causes less variation in maternal mean blood pressure and heart rate than thiopental during rapid sequence induction of anesthesia for a selective cesarean section and has no adverse effects on neonates. The promising results warrant further investigation into the use of propofol in obstetric surgery	29.8 ± 5.2 32.9 ± 3	80 ± 9 86.2 ± 11.3	38.2 ± 0.9 38.1 ± 0.6	NA
So et al., 1996 [45]	Korea	1)Thiopental (20) 2)Propofol(20)	1) 4:5 2) 2	Apgar score, Umbilical venous gas	1)enflurane and nitrous oxide 2)propofol and nitrous oxide	to compare Apgar scores, maternal cardiovascular parameters, and recovery time between thiopental and propofol-induced anesthesia	At 1 min after intubation, systolic and mean arterial pressure and HR were significantly increased but the level was less in the propofol group. There was no significant difference as regards DBP, umbilical blood gases, and APGAR score	30.5 ± 4.5 31.3 ± 3.7	66.5 ± 8.6 71.3 ± 9.3	NA	NA
Tumukunde et al., 2015 [7]	Uganda	1)Propofol (75) 2)Thiopental (75)	1) 2 2) 4	Apgar score	isoflurane + O2	to investigate the effects of thiopental and propofol on the neonatal Apgar score and maternal recovery time following cesarean section	the Apgar score did not differ significantly between propofol and thiopental groups. Propofol has a significantly shorter maternal recovery time	25.16 ± 5.11 24.017 ± 6.11	58.56 ± 9.31 66.67 ± 9.68	NA	NA
Valtonen et al., 1989 [12]	Finland	1)Propofol (16) 2)Thiopental (16)	1) 2.5 2) 5	Apgar score and UBGs	isoflurane + N2O + O2	to compare propofol with thiopentone for induction of anesthesia in elective CS. Particular attention was paid to induction characteristics and the neonatal effects(umbilical blood gases and APGAR score) of both agents	Propofol is similar to thiopental in induction characteristics and effects on neonates. Propofol shows shorter recovery times. There was no significant neonatal depression as assessed by Apgar scores and blood gas analyses. Propofol appears to be a suitable alternative to thiopental	27.4 ± 2.85 30.5 ± 5.46	79.1 ± 8.94 75.9 ± 8.87	NA	NA
Wanna et al., 2004 [6]	Thailand	1)Propofol (50) 2)Ketamine (50)	1) 2 2) 1	Apgar score and maternal cardiovascular responses	sevoflurane + O2 + N2O	to evaluate neonatal and maternal effects of propofol and ketamine as induction drugs for elective cesarean section	Ketamine and propofol can be used as alternatives for thiopental with no significant neonatal effect as measured by Apgar scores after 1 min. No awareness, low incidence of dreams and postoperative nausea & vomiting (PONV), and low incidences of unpleasant sleep in the mother were found	28.54 ± 5.89 27.16 ± 4.73	65.55 ± 9.6 63.26 ± 9.12	NA	NA
Yau et al., 1991 [46]	China	1)Thiopental (20) 2)Propofol(20)	1) 4 2) 2	Apgar score and UBGs	1)enflurane 2)propofol	to compare a propofol infusion plus nitrous oxide with a thiopentone, enflurane, and nitrous oxide general anesthetic	Both thiopental and propofol provided satisfactory maternal and neonatal outcomes, with rapid maternal recovery. However, prolonged propofol infusion before delivery may slightly lower neonatal Neurologic and Adaptive Capacity Scores	30.6 ± 5.1 29.4 ± 4.3	65.4 ± 9.4 65.8 ± 9.3	38.9 ± 1.3 39.1 ± 1.5	NA
Schultetus et al., 1985 [44]	USA	1)Thiopentone(32) 2)Ketamine(30)	1) 4 2) 1	Hemodynamic changes, Apgar score, and UBG	succinylcholine + N2O + O2	compare between Hemodynamic effects of ketamine and thiopentone during anesthetic induction for Cesarean section	For normotensive pregnant patients, ketamine provides cardiovascular stability; neonatal outcomes are good; and the incidences of maternal awareness and emergence phenomena are low. Therefore, they think ketamine is an acceptable and useful alternative to thiopentone to induce anesthesia for Caesarean section	23.5 ± 5.6 22.8 ± 4.9	75.1 ± 12.9 75.3 ± 13.7	NA	NA
Rabiee et al., 2012 [47]	Iran	1)Thiopental(25) 2)Propofol(25)	1) 5 2) 2.5	BIS and Apgar score	Isofluran + NO + O2	This study is designed to compare sodium thiopental and propofol as induction agents in Depth of anesthesia and hemodynamic variations in mothers and APGAR score of neonates	The effect of sodium thiopental and propofol on depth of anesthesia and hemodynamic variables of mothers as well as neonatal APGAR scores was similar and propofol can be used as an appropriate alternative for sodium thiopental in induction of anesthesia for cesarean section	26.32 ± 4.31 27.6 ± 5.04	NA	NA	NA
Nayar et al., 2009 *5 [48]	India	1)Thiopental(20) 2)Ketamine (20)	1) 5 2) 1	Hemodynamic Changes	N2O + O2 + halothane	To evaluate the benefit of a combination of thiopentone and ketamine over either of these drugs alone as an induction agent for Cesarean section	The use of ketamine alone as an induction agent in Cesarean section should be avoided, as it causes significant maternal hemodynamic changes. The addition of a reduced dose of ketamine to thiopentone in the induction cocktail confers the benefit of reducing analgesic requirements without side effects. The treatment is safe and effective for the mother and child	24.6 ± 4.3 23.3 ± 4.1	57.6 ± 5.85 57.2 ± 7.17	NA	NA
Gregory et al., 1990 *6 [49]	China	1)Thiopentone(10) 2)Propofol low infusion(10)	1) 4 2) 2	Maternal hemodynamics and Apgar score	1)Enflurane + N20 + 02 2)Propofol + N2O + 02	To compare thiopentone with propofol as regards hemodynamics and Apgar score as an induction agent for CS	Hemodynamic changes were similar immediately following induction. Neonatal Apgar scores and umbilical blood gas analysis were similar	28.4 ± 4.6	62.6 ± 8.7	NA	NA
Krissel et al., 1989*7 [51]	Germany	1)Thiopentone(25)2)Ketamine(25)	1)4 2)1	UBG and Apgar score	1)Enflurane and N2O 2)N2O and ketamine	To compare thiopentone, ketamine, and a combination of them with regard to UBGs, Apgar score, and awareness	The number of neonates with APGAR score less than seven at one and five minutes was higher in ketamine group and only one patient in thiopentone group experienced awareness	NA	NA	NA	NA

^*^1 We presented this study as Baraka 1 for maintenance 1 and Baraka 2 for maintenance; in addition to this, This study included a further treatment group of 10 patients who received ketamine 1.5 mg kg − 1 for induction and 100% oxygen for maintenance

^*^2 This study included a further group of 44 patients who received methohexitone 1 mg/kg for induction of anesthesia

^*^3 This study included a further group of 15 patients who received propofol 1 mg/kg and ketamine 0.5 mg/kg for induction of anesthesia

^*^4 This study had a further treatment group of 11 patients who received a combination of thiopentone 2 mg/kg and ketamine 0.5 mg/kg for induction

^*^5 This study had a further treatment group of 20 patients who received a combination of thiopentone 2.5 mg/kg and ketamine 0.5 mg/kg for induction

^*^6 This study had a further treatment group of 10 patients who received propofol 2 mg/kg for induction and propofol 9 mg/kg with 100% O2 for maintenance

^*^7 This study included a further group of 25 patients received a combination of thiopentone(2 mg/kg) and ketamine(0.5 mg/kg) for induction of anesthesia

### Quality assessment

**Fig. 2:**
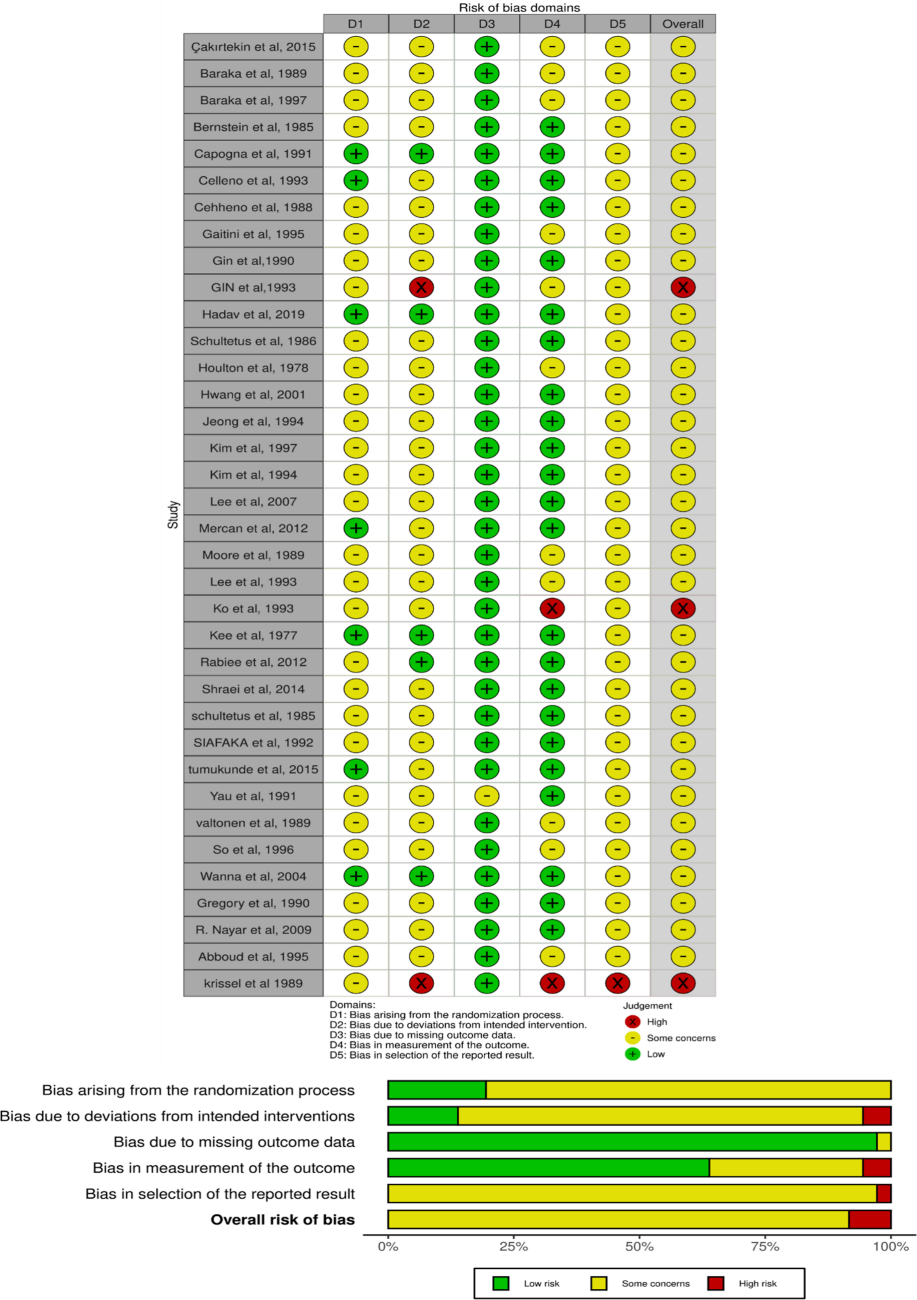
A Risk of Bias analysis of the included studies in our systematic review and meta-analysis.” + ” indicates low risk;”-” indicates high risk and”!” indicates some concerns

The quality assessment was conducted using the revised Cochrane risk-of-bias tool for randomized clinical trials (ROB 2). Out of 36 studies, three had a high risk of bias, the rest of the studies had some concerns (Fig. 2).

### Analysis

#### Quantitative analysis of studies comparing thiopentone with propofol

Twenty-four studies compared thiopental versus propofol. Most of them reported the following outcomes: Apgar score, umbilical artery and vein blood gases (PO_2_, PCO_2_, pH, HCO_3_, BE, and SPO_2_), maternal blood gases (PO_2_, PCO_2_, pH, and BE), recovery duration, and incision to delivery time. The overall quality of evidence is moderate to very low (Table 2)**.**

**Table 2:** Summary of Findings (SoF) for the comparisons between Thiopental and Propofol, and Thiopental and Ketamine

Outcomes	Number of studies	Number of patients		Effect		Certainty
		Comparison (Propofol or Ketamine)	Thiopental	Relative (95% CI)	Absolute (95% CI)	
*Comparison of Thiopental versus Propofol*						
pH in UA	12	263	266	-	**0** (0.01 lower to 0)	⨁⨁⨁◯ Moderate^a,f^
Apgar score < 7 at one minute	7	81/230 (35.2%)	43/230 (18.7%)	**RR 2.06** (1.06 to 4.01)	**198 more per 1,000** (from 11 to 563 more)	⨁◯◯◯ Very low^a,b,c,d^
Apgar score < 7 at five minutes	7	15/230 (6.5%)	8/230 (3.5%)	**RR 1.75** (0.81 to 3.78)	**26 more per 1,000** (from 7 fewer to 97 more)	⨁⨁◯◯ Low^a,c,d,e^
Recovery duration	4	91	91	-	MD **112.36 mmHg lower** (153.36 lower to 71.61 lower)	⨁⨁⨁◯ Moderate^a,f^
BE in UA	8	177	175	-	MD **0.55 mmHg higher** (0.08 lower to 1.17 higher)	⨁◯◯◯ Very low^a,b,e^
in HCO_3_ in UA	3	78	80	-	MD **1.25 mmHg lower** (3.44 lower to 0.93 higher)	⨁◯◯◯ Very low^a,b,c,d,e^
SPO_2_ in UV	5	149	152	-	MD **0.5 mmHg lower** (6.7 lower to 5.7 higher)	⨁◯◯◯ Very low^a,b,c,d,e^
*Comparison of Thiopental versus Ketamine*						
PvO_2_ in UV	9	179	174	-	MD **0.23 mmHg higher** (0.02 higher to 0.45 higher)	⨁⨁⨁◯ Moderate^a,f^
Apgar score < 7 at 1 min	9	35/170 (20.6%)	18/175 (10.3%)	**RR 0.52** (0.31 to 0.85)	**49 fewer per 1,000** (from 71 to 15 fewer)	⨁⨁⨁◯ Moderate^a,f^
Apgar score < 7 at 5 min	9	13/170 (7.6%)	3/175 (1.7%)	**RR 0.28** (0.10 to 0.79)	**12 fewer per 1,000** (from 15 to 4 fewer)	⨁⨁⨁◯ Moderate^a,f^
AAGA	8	9/148 (6.1%)	29/147 (19.7%)	**RR 2.87** (1.58 to 5.21)	**369 more per 1,000** (from 114 to 831 more)	⨁⨁⨁◯ Moderate^a,f^
pH in UA	4	66	65	-	MD **0 mmHg** (0.02 lower to 0.01 higher)	⨁⨁◯◯ Low^a,c,e^
pH in UV	5	86	85	-	MD **0 mmHg** (0.02 lower to 0.02 higher)	⨁⨁◯◯ Low^a,c,e^
PO₂ in UA	6	141	135	-	MD **0.04 mmHg higher** (0.12 lower to 0.2 higher)	⨁⨁◯◯ Low^a,c,e^

*CI* confidence interval, *MD* mean difference, *RR* risk ratio

a. Downgraded due to risk of bias in studies (most studies have some concerns, and three studies of high risk)

b. Downgraded due to inconsistency (High heterogeneity)

c. Downgraded due to imprecision (Small sample size)

d. Downgraded due to imprecision (Wide confidence interval)

e. Downgraded due to imprecision (Confidence interval includes both benefit and harm)

f. The confidence interval is narrow and supports a clear direction of effect

**Fig. 3:**
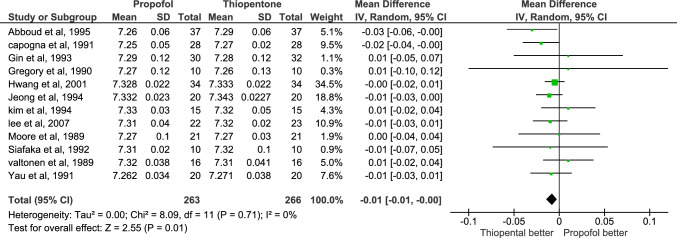
Forest plot of mean difference in pH in UA, thiopentone vs. Propofol

Twelve studies reported pH in UA with 529 patients; 263 in the propofol group, and 266 in thiopentone group. It was higher in thiopentone group with MD = − 0.01, and P-value = 0.01. There was no heterogeneity among studies (I2 = 0%), and the certainty of evidence is Moderate Fig. 3.

**Fig. 4:**
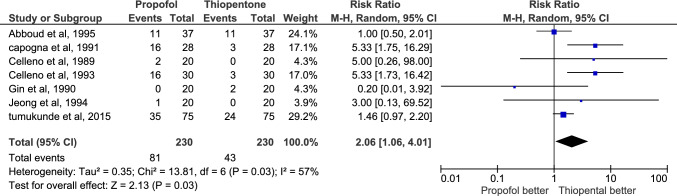
Comparison of the number of newborns with Apgar score < 7 at 1 min after delivery, thiopentone vs. propofol

**Fig. 5:**
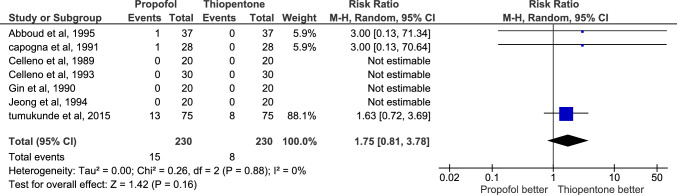
Comparison of the number of newborns with Apgar score < 7 at 5 min after delivery, thiopentone vs. propofol

Apgar score < 7 at one and five minutes was reported by 7 studies. The total number of neonates was 230 for each group. 81 neonates had a score < 7 at one minute in propofol group, and is higher than thiopental group, which had 43 neonates less than 7 in thiopentone group and the certainty of evidence was very low. At 5 min, 15 neonates in the propofol group had Apgar score < 7 and their number was only 8 in thiopentone group, but the certainty of evidence was low. At one minute, the risk ratio was 2.06 (CI 1.06:4.01) with P-value = 0.03; but at five minutes, the risk ratio was 1.75(CI 0.81:3.78) with P-value = 0.16. Heterogeneity was tested and I2 was 57% at one minute and 0% at five minutes indicating non-significant heterogeneity Fig. 4, 5.

**Fig. 6:**
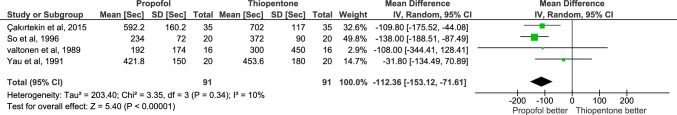
Forest plot of mean difference in Recovery duration, thiopentone vs. propofol

Recovery duration was reported by 4 studies with a total number of patients 182 patients, 91 in each group. It was longer in thiopentone than in the propofol group with MD = -112.36, P-value < 0.00001, and the certainty of evidence was moderate. Heterogeneity among groups was not significant(I2 = 10%) Fig. 6.

The rest of the umbilical blood gases, maternal blood gases, and other outcomes did not differ significantly among groups (Supplementary Figs. 4 to 22 and 42 to 44).

Based on current data, TSA showed that further trials with at least 346 and 3,010 patients are needed to confirm reliable results for Apgar score < 7 at one minute and umbilical artery Po₂, respectively. These numbers may change with future studies depending on effect size, study quality, and heterogeneity (Supplementary Figs. 46 and 47).

#### Quantitative analysis of studies comparing thiopentone with ketamine:

Twelve studies compared thiopental versus ketamine. The analysis was done in the following outcomes: Apgar score, umbilical artery and vein blood gases (PO_2_, PCO_2_, and pH), maternal blood gases (PO_2_, PCO_2_), dreams, recall, AAGA, and uterine incision delivery time. The overall quality of evidence was moderate to low.

**Fig. 7:**
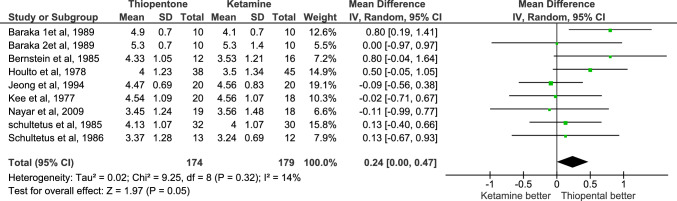
Forest plot of mean difference in PO_2_ in UV, thiopentone vs. ketamine

PvO_2_ in UV was reported in 8 studies with a total number of patients equal to 353, 174 in the thiopentone group and 179 in the ketamine group. It was higher in thiopentone group with MD = 0.24, and P-value = 0.05. Heterogeneity was not significant (I2 = 14%),and the certainty of evidence was moderate Fig. 7.

**Fig. 8:**
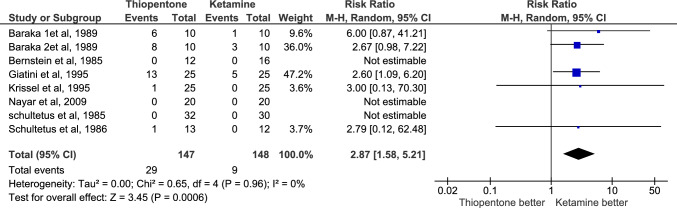
Comparison of number of infants having Apgar score < 7 at 1 min after delivery, Thiopentone vs. Ketamine

**Fig. 9:**
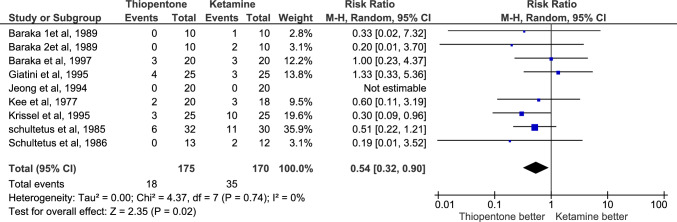
Comparison of number of infants having Apgar score < 7 at 5 min after delivery, Thiopentone vs. Ketamine

Apgar score < 7 at 1 and 5 min were reported by 9 studies with a total number of patients equal to 345, 175 in the thiopentone group and 170 in the ketamine group. Having an infant with an Apgar score < 7 at 1 and 5 min after delivery is significantly less frequent in the thiopentone group (n = 18 and 3 respectively) than in the ketamine group(n = 35 and 13 respectively); RR = 0.54, P-value = 0.02 and 0.29, P-value = 0.02 respectively. There was no heterogeneity among reporting studies; I^2^ = 0% for both outcomes, and the certainty of evidence for both outcomes was moderate Fig. 8, 9.

**Fig. 10:**
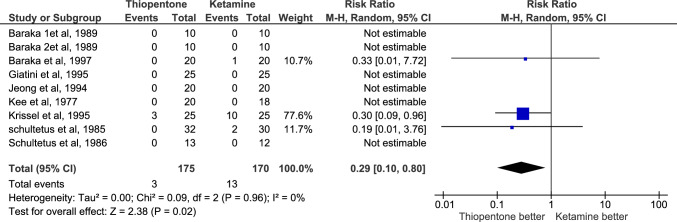
Comparison of number of patients suffered from AAGA, Thiopentone vs. Ketamine

AAGA was reported by 7 studies with 295 patients, 147 in the thiopentone group and 148 in the ketamine group. It was more prevalent in thiopentone (n = 29) than in ketamine group (n = 9). RR = 2.87, and P-value = 0.0006. There was no heterogeneity among studies (I2 = 0%), and the certainty of evidence was moderate Fig. 10**.**

The rest of umbilical blood gases, and other outcomes did not differ significantly among groups (Supplementary Figs. 23 to 32).

#### Quantitative analysis of studies comparing Propofol with ketamine:

Three studies compared propofol versus ketamine. Most of them reported the following outcomes: Apgar score, umbilical artery and vein blood gases (PO_2_, PCO_2_, and pH), and uterine incision delivery time. There was no significant difference between groups concerning these outcomes (Supplementary Fig. 33–41). The overall quality of evidence was very low (GRADE assessment supplementary table).

### Leave one out a meta-analysis

We conducted leave-one-out analyses to solve the heterogeneity between studies and to assess the strength of the results.

The leave-one-out meta-analysis showed no significant difference in heterogeneity for all analyses except for 3 analyses in the comparison of thiopentone vs propofol and one analysis in the comparison of thiopentone vs ketamine;

in removing Capogna et al., 1991 from the BE and HCO_3_ in UA analysis, heterogeneity changed significantly from I2 = 64% and I2 = 88% respectively to I2 = 0% for both. The MD in both outcomes didn’t change significantly; MD was 0.55; P-value = 0.09 and -1.25; P-value = 0.26 and changed to 0.24; P-value = 0.16 and -0.16; P-value = 0.73 respectively. (Supplementary Fig. 1–2).

In addition, after removing Lee et al., 2007 from SPO_2_ in UV analysis, heterogeneity significantly changed from I2 = 75% to I2 = 42% but MD remained non-significant; it was -0.5; P-value = 0.87 and became 2.25; P-value = 0.34 after re-analysis (Supplementary Fig. 3).

Also, after removing Krissel et al., 1989 from the analysis s of the Apgar score at 5 min in (Thiopentone vs. Ketamine) group, heterogeneity was not affected but the risk ratio became insignificant. It was 0.25(CI 0.03:2.16) and became 0.28(CI 0.1:0.79) (Supplementary Fig. 45).

## Discussion

We conducted this updated systematic review and meta-analysis as an update of the Khemlani et al. (2018) study [23]; Several methodological enhancements distinguish our study from the previous review, which strengthens the evidence base. Concerning literature search, while the earlier study searched PubMed and the Cochrane Library, we broadened our search to also include Web of Science and Scopus. Our search extended through August 2024, compared to January 2017 in the previous review. As a result, we included 36 studies involving 1,945 patients, in contrast to the 18 studies and 911 patients analyzed by Khemlani et al. Notably, we identified 14 eligible studies from the same time frame as the original review that were previously missed.

Also, our analysis improved in many outcomes; Instead of comparing absolute systolic blood pressure (SBP) values, we analyzed the difference in changes, which offers greater statistical accuracy. Additionally, we examined changes in diastolic blood pressure (DBP) and heart rate (HR), which were not addressed previously. We also included a new comparison between propofol and ketamine, which was absent in the earlier review; We assessed several novel outcomes such as SpO₂ and HCO₃ levels in uterine arteries and veins, the presence of dreams or recall, and the durations of anesthesia and recovery. Furthermore, unlike the prior review which only described AGAA outcome qualitatively, we conducted a quantitative analysis of AAGA.

There is outcomes in our study showed statistically significant differences compared to the previous review. Specifically, significant differences were observed in pH in the umbilical artery and in the risk of an Apgar score < 7 at 1 min outcomes when comparing thiopentone and propofol, as well as in PvO_2_ in the UV when comparing thiopentone and ketamine. Conversely, our findings for PaO_2_ in the umbilical artery in the thiopentone versus propofol comparison were not statistically significant, despite previous reports indicating significance. Thus, our study presents considerably stronger evidence across multiple outcomes, as supported by the GRADE assessment of the evidence quality.

### Thiopental and propofol

The results of our study showed that there was no significant difference between thiopental and propofol groups except for the UA pH, APGAR score at 1 min, and recovery time. pH in UA was significantly higher in thiopentone group than the propofol group with MD = − 0.01(CI − 0.01:0.00) and P-value = 0.01. The Apgar score was reported as mean ± SD at one and five minutes in 16 and 17 studies, respectively, with no significant differences observed between study groups at either time point. Additionally, seven studies reported the number of neonates with an Apgar score < 7 at one and five minutes; Induction with propofol was associated with higher neonates having an Apgar score < 7 at one minute compared to thiopental. However, the clinical relevance of this finding remains questionable, especially given the lack of difference at five minutes.

Compared to induction with thiopental, induction with propofol showed a significantly shorter recovery duration with a mean difference of − 12.36 [− 153.12, − 7161] (Fig. 6). This could be explained by the lipophilic nature of propofol and its rapid metabolism by the liver; The pharmacokinetic profile of propofol may give it an advantage in outpatient anesthesia [52, 53]. Some studies also concluded propofol is more advantageous than thiopental as it provides adequate anesthetic depth and rapid recovery. [30]

### Thiopental and ketamine

Regarding thiopental and ketamine groups, no significant difference was found except for the UV PvO_2_, AAGA, and Apgar score. The PvO_2_ in UV was significantly higher in the thiopentone group with MD = 0.23 and P-value = 0.04.

Accidental awareness was more common in the thiopental group compared to the ketamine group, with an RR of 2.87 (95% CI 1.58 to 5.21). In our study, AAGA in thiopental and ketamine was about 20% and 6% respectively which is consistent with the fifth National Audit Project (NAP5), which reported AAGA incidences to be about 23% and 5% in thiopental and ketamine respectively [54].

The cause of this difference is not exactly known but may be attributed to the shorter duration of action of thiopental compared to ketamine, which could increase the risk of AAGA if not properly managed with a maintenance agent. In contrast, ketamine is relatively potent in providing analgesia and amnesia, offering better protection against AAGA [55, 56].

Regarding the APGAR score, in most studies, the number of infants with an Apgar score < 7 at 1 min was higher in the ketamine group than in the thiopentone group [14, 22, 42, 44, 51]. Both groups were equal in the two studies [28, 35] and the thiopentone group had more cases in just one study [50]. Apgar score < 7 at 5 min after delivery was more frequent in the ketamine group in 3 studies[28, 44, 51] and equal between both groups in 5 studies [14, 22, 35, 42, 50].

Although the difference is statistically significant, its clinical relevance remains uncertain due to the relatively small differences between the groups. An exception is Krissel et al., 1989, in which the difference became statistically insignificant when this study was excluded in the leave-one-out meta-analysis. These variations may be explained by differences in study design, dosages, patient populations, and clinical settings. Thus, Further well-designed, large-scale studies are required to better understand the impact of these induction agents on neonatal Apgar scores.

### Propofol and ketamine

Three studies comparing propofol and ketamine in a total of 170 patients found no significant differences in any reported outcomes, although the quality of evidence is low due to the limited number of studies and small sample size. Pharmacologically, both agents are rapid-acting intravenous drugs with a short duration of action because of their high lipid solubility and rapid redistribution, enabling fast induction and smooth recovery. Despite working through different mechanisms—propofol by enhancing GABA_A receptor activity and ketamine by blocking NMDA receptors—their overall clinical profiles for induction are comparable. [57–60]

### Limitations and recommendations

The study has some limitations. The variation in methodologies across the studies, the differing outcome measures used, and the small sample sizes of some studies may have influenced bias and the overall quality of evidence. This heterogeneity was partly addressed through sensitivity analyses, leave-one-out meta-analyses, and the application of a random-effects model. The diverse approaches used to assess intraoperative awareness are also important to consider. Additionally, a significant limitation is that many included studies were conducted in earlier periods, particularly between the 1980s and early 2000s, during which clinical protocols, perioperative monitoring, and equipment differed considerably from current standards. For example, modern devices such as videolaryngoscopes, supraglottic airway devices, and monitoring tools like capnography and pulse oximetry are now considered standard practice [61–63]. These advancements may influence maternal and neonatal outcomes, such as neonatal resuscitation. This may limit the applicability of older studies to present-day clinical settings. In addition, we didn’t have access to the EMBASE database, which was included in the previous review; however, we attempted to address this by broadening our search to include Web of Science and Scopus to enhance the comprehensiveness of our literature retrieval. Another limitation is the lack of high-quality studies in the analysis. Therefore, more recent and well-designed randomized controlled trials are needed to provide stronger, more generalizable evidence.

## Conclusion

This systematic review revealed that the use of propofol was associated with higher neonates with APGAR scores less than 7 at 1 min compared to thiopental. Accidental awareness was also higher among the thiopental group than among the ketamine group. Included studies showed variation in methodologies and outcome measures, which may have impacted bias and evidence quality. Additional well-designed trials are needed to more firmly support our conclusions.

## Supplementary Information

Below is the link to the electronic supplementary material.

Supplementary file1 (DOCX 1582 KB)

Supplementary file2 (DOCX 21 KB)

Supplementary file3 (PDF 150 KB)

## Data Availability

NA.

## Abbreviations

PaO₂: Partial arterial pressure of oxygen in the blood
PvCO₂: Partial venous pressure of carbon dioxide in the blood
BE: Base Excess
SpO₂: Peripheral capillary oxygen saturation
HCO₃: Bicarbonate level in the blood
UV: Umbilical vein
UA: Umbilical artery
AAGA: Accidental awareness during general anesthesia
pH: Potential of hydrogen
ROB 2: Risk of Bias 2
