# Supplementary material for: Hypnotics as induction agents for general anesthesia in cesarean section patients: updated systematic review and meta-analysis of randomized controlled trials
Source: J Anesth. 2025 Jun 21;39(6):948–75. doi: 10.1007/s00540-025-03524-8 (PMC12647321; doi:10.1007/s00540-025-03524-8)
Supplement: Supplementary file 1 — Supplementary file1 (DOCX 1582 KB) [file 540_2025_3524_MOESM1_ESM.docx]

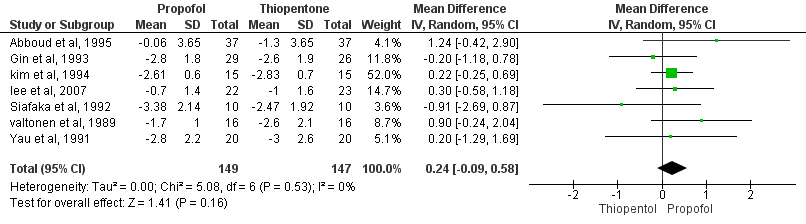


*Supplementary Figure 1: Leave one out meta-analysis of BE in UA after exclusion of Capogna et al, 1991, thiopentone vs. propofol.*


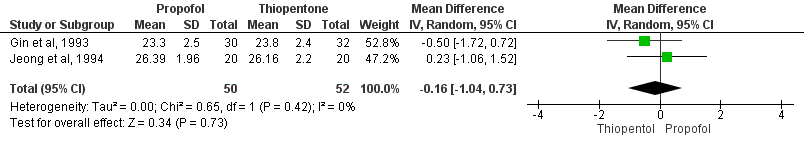


*Supplementary Figure 2: Leave one out meta-analysis of Hco3 in UA after exclusion of Capogna et al, 1991, thiopentone vs. Propofol.*


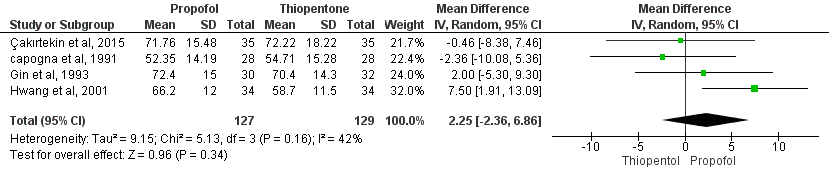


*Supplementary Figure 3: Leave one out meta-analysis of Spo2 in UV after the exclusion of Lee et al, 2007, thiopentone vs. propofol.*


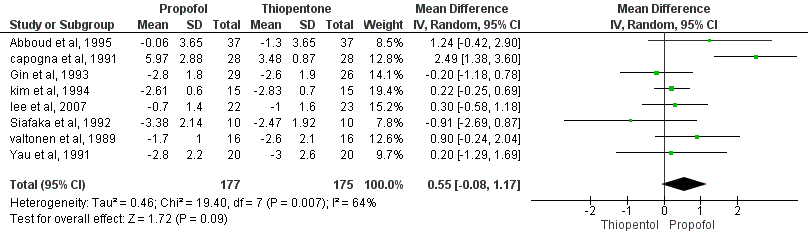


*Supplementary Figure 4: Forest plot of mean difference in BE in UA, thiopentone vs. propofol.*


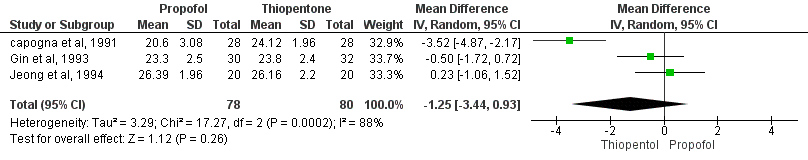


*Supplementary Figure 5: Forest plot of mean difference in Hco3 in UA, thiopentone vs. propofol.*


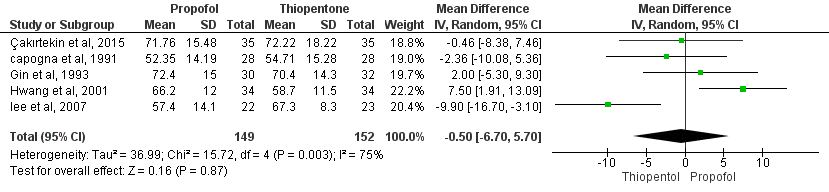


*Supplementary Figure 6: Forest plot of mean difference in Spo2 in UV, thiopentone vs. propofol.*


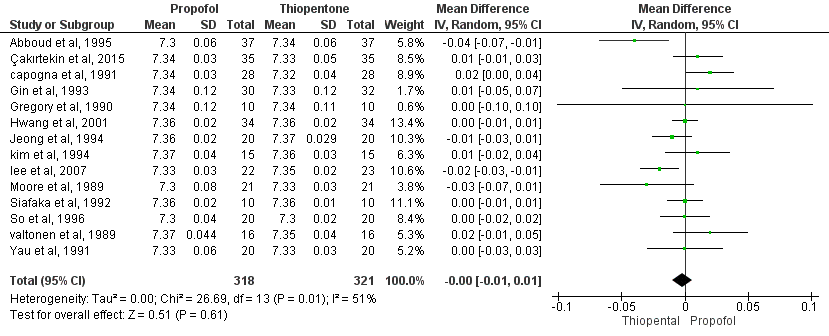


*Supplementary Figure 7: Forest plot of mean difference in PH in UV, thiopentone vs. propofol.*


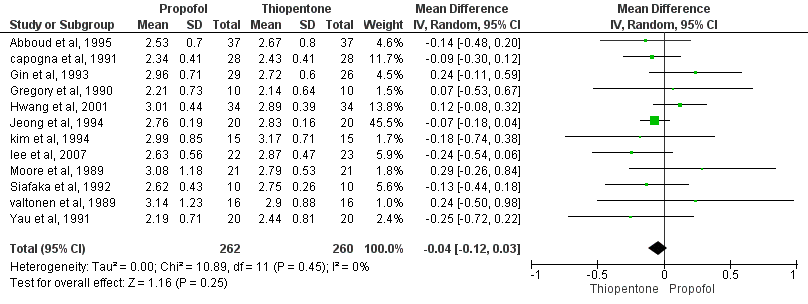


*Supplementary Figure 8: Forest plot of mean difference in Po2 in UA, thiopentone vs. propofol.*


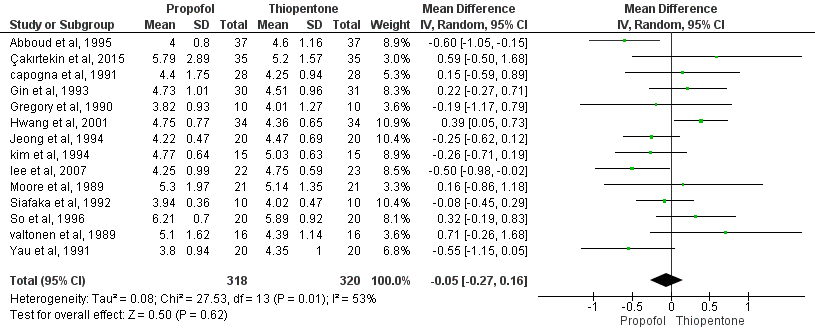


*Supplementary Figure 9: Forest plot of mean difference in Po2 in UV, thiopentone vs. Propofol.*


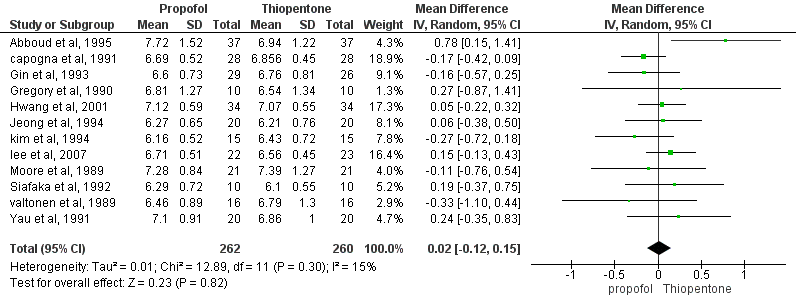


*Supplementary Figure 10: Forest plot of mean difference in Pco2 in UA, thiopentone vs. Propofol.*


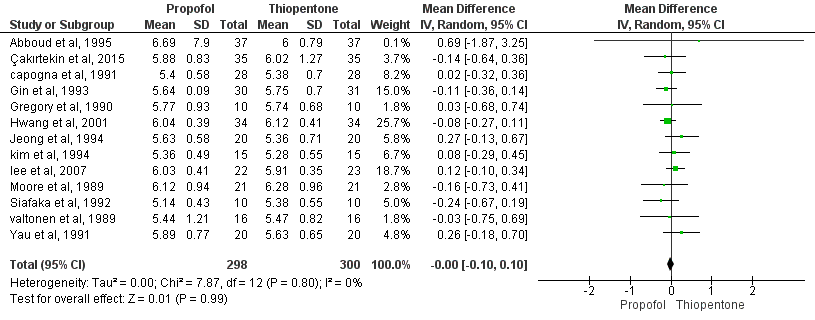


*Supplementary Figure 11: Forest plot of mean difference in Pco2 in UV, thiopentone vs. propofol.*


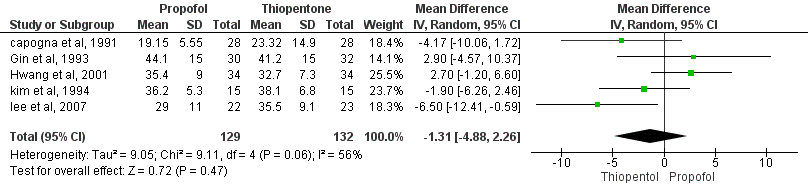


*Supplementary Figure 12: Forest plot of mean difference in Spo2 in UA, thiopentone vs. propofol*


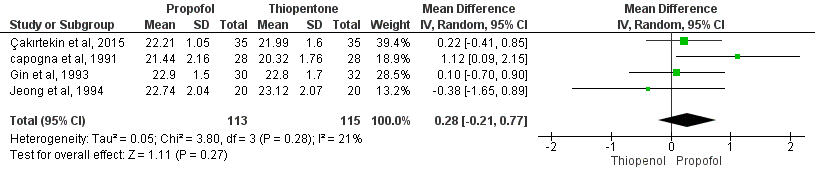


*Supplementary Figure 13: Forest plot of mean difference in Hco3 in UV, thiopentone vs. propofol.*


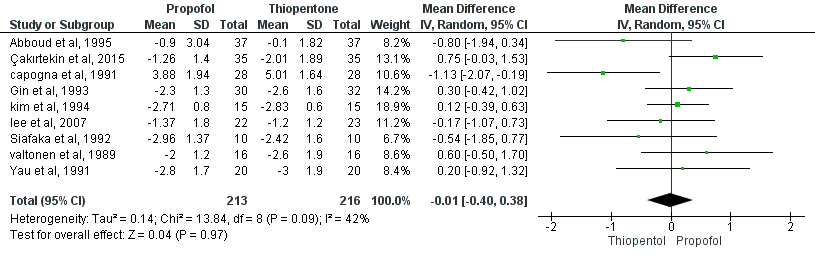


*Supplementary Figure 14: Forest plot of mean difference in BE in UV, thiopentone vs. propofol.*


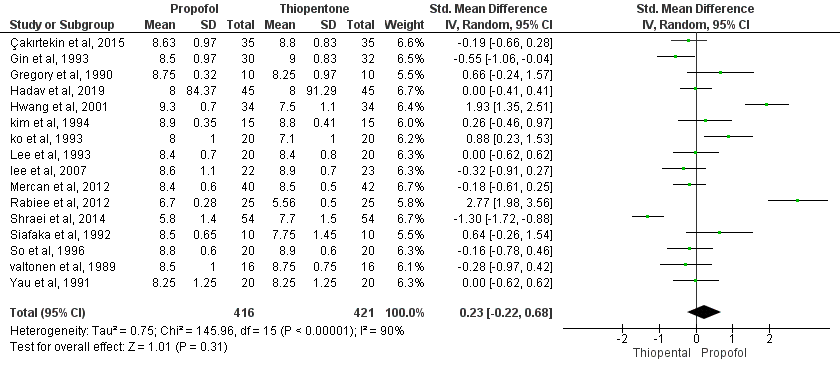


*Supplementary Figure 15: Forest plot of mean difference in Apgar score at one minute, thiopentone vs. propofol.*


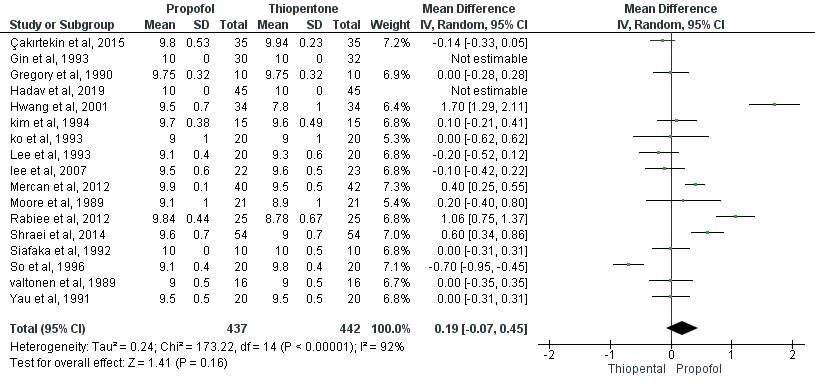


*Supplementary Figure 16: Forest plot of mean difference in Apgar score at five minutes, thiopentone vs. propofol.*


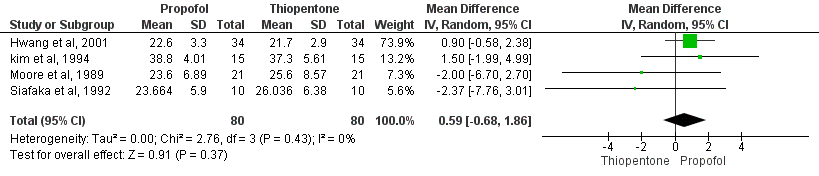


*Supplementary Figure 17: Forest plot of mean difference in Po2 in maternal blood, thiopentone vs. propofol.*


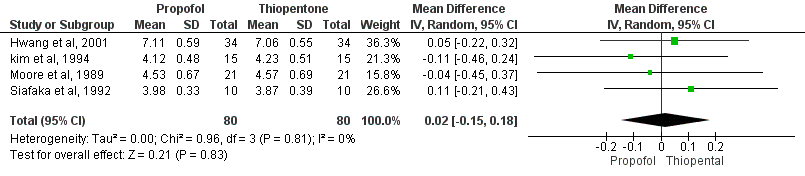


*Supplementary Figure 18: Forest plot of mean difference in Pco2 in maternal blood, thiopentone vs. propofol.*


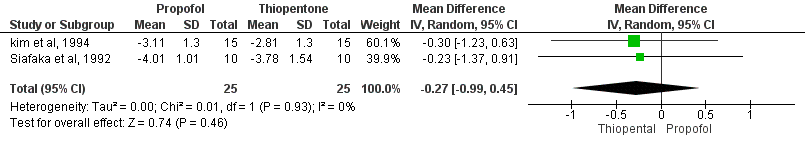


*Supplementary Figure 19: Forest plot of mean difference in BE in maternal blood, thiopentone vs. Propofol.*


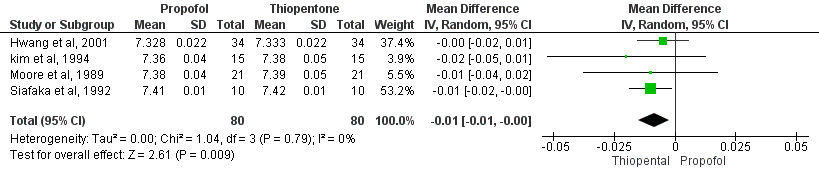


*Supplementary Figure 20: Forest plot of mean difference in PH in maternal blood, thiopentone vs. Propofol.*


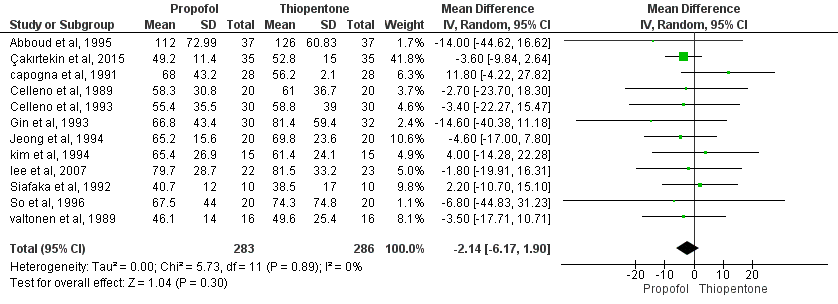


*Supplementary Figure 21: Forest plot of mean difference in Uterine incision to delivery time, thiopentone vs. propofol.*


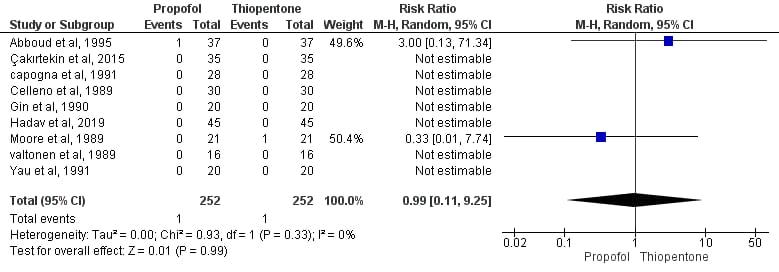


*Supplementary Figure 22: Comparison of number of patients suffering from AAGA, thiopentone vs. propofol.*


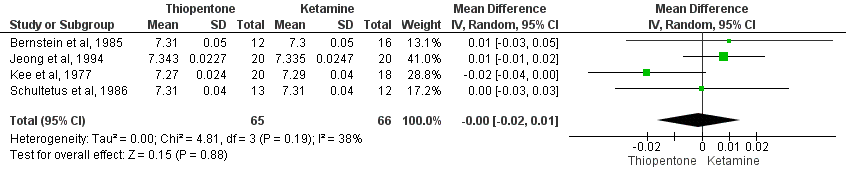


*Supplementary Figure 23: Forest plot of mean difference in PH in UA, thiopentone vs. ketamine.*


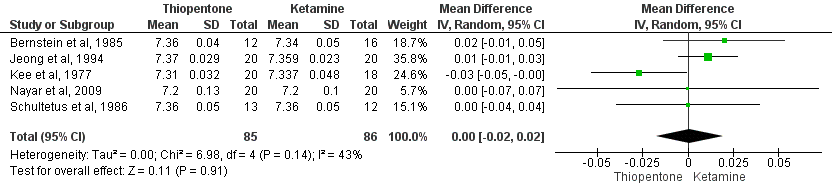


*Supplementary Figure 24: Forest plot of mean difference in PH in UV, thiopentone vs. ketamine.*


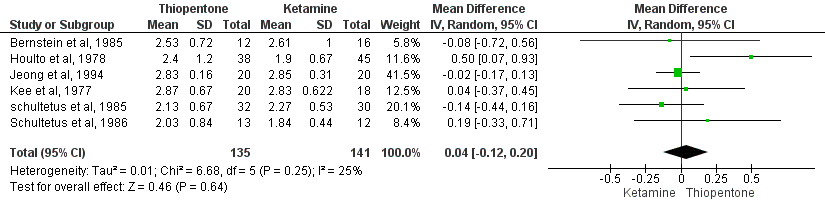


*Supplementary Figure 25: Forest plot of mean difference in Po2 in UA, thiopentone vs. ketamine.*


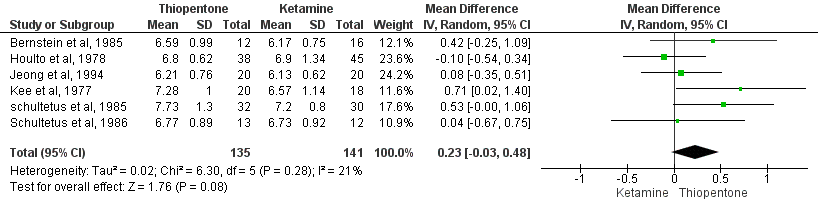


*Supplementary Figure 26: Forest plot of mean difference in Pco2 in UA, thiopentone vs. ketamine.*


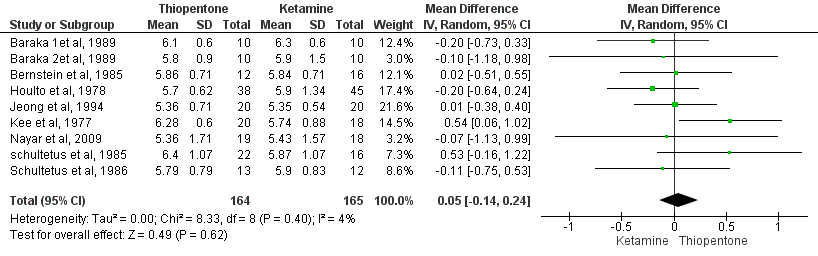


*Supplementary Figure 27: Forest plot of mean difference in Pco2 in UV, thiopentone vs. ketamine.*


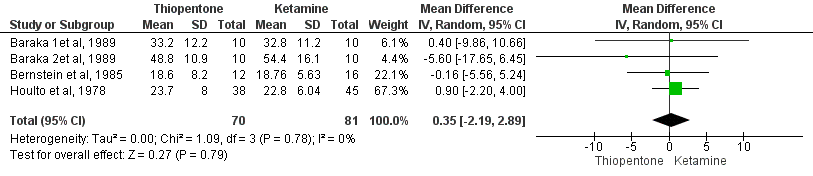


*Supplementary Figure 28: Forest plot of mean difference in Po2 in maternal blood, thiopentone vs. ketamine.*


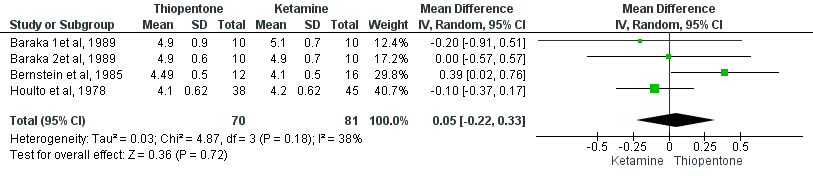


*Supplementary Figure 29: Forest plot of mean difference in Pco2 in maternal blood, thiopentone vs. ketamine.*


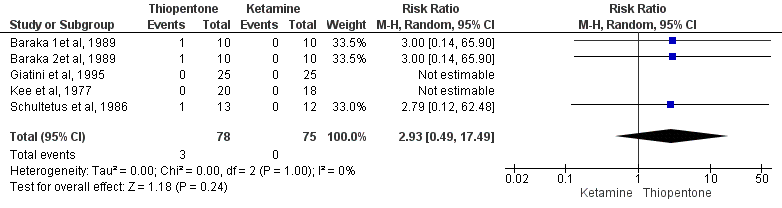


*Supplementary Figure 30: Comparison of the number of patients who experienced recall, thiopentone vs. ketamine.*

*
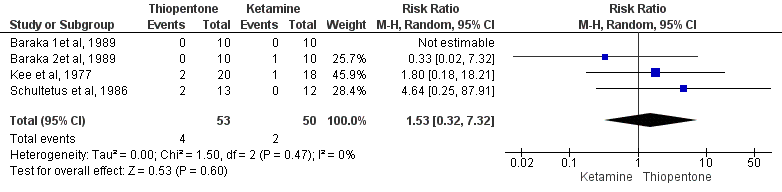
*

*Supplementary Figure 31: Comparison of the number of patients suffering from dreams, thiopentone vs. ketamine.*


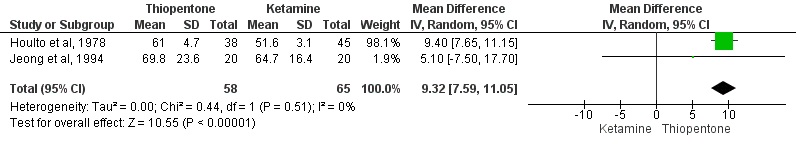


*Supplementary Figure 32: Forest plot of mean difference in Uterine incision to delivery time, thiopentone vs. ketamine.*


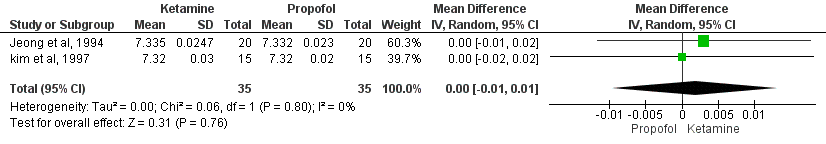


*Supplementary Figure 33: Forest plot of mean difference in PH in UA, propofol vs. ketamine.*


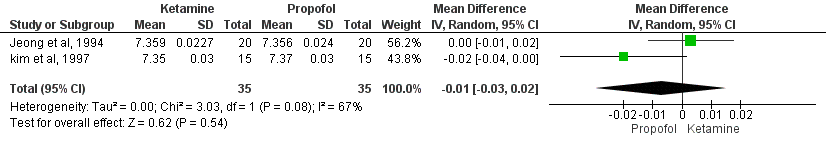


*Supplementary Figure 34: Forest plot of mean difference in PH in UV, propofol vs. ketamine.*


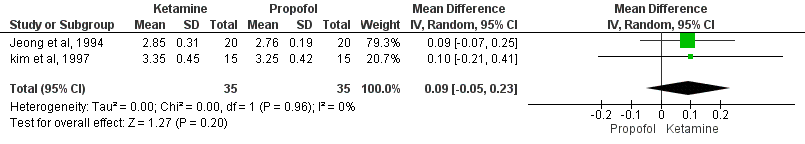


*Supplementary Figure 35: Forest plot of mean difference in Po2 in UA, propofol vs. ketamine.*


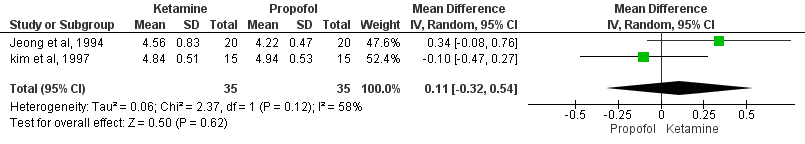


*Supplementary Figure 36: Forest plot of mean difference in Po2 in UV, propofol vs. ketamine.*


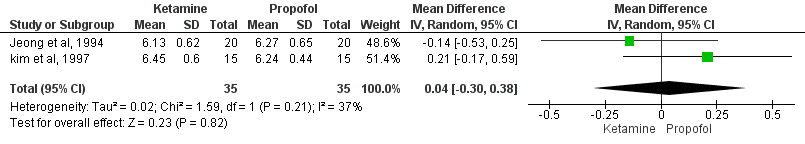


*Supplementary Figure 37: Forest plot of mean difference in Pco2 in UA, propofol vs. ketamine.*


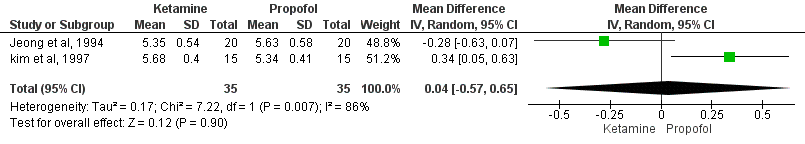


*Supplementary Figure 38: Forest plot of mean difference in Pco2 in UV, propofol vs. ketamine.*


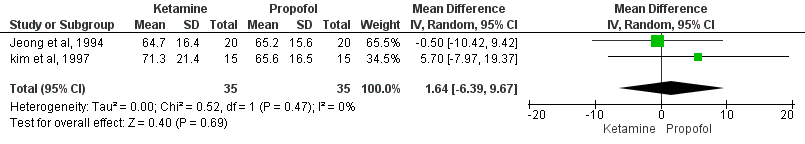


*Supplementary Figure 39: Forest plot of mean difference in uterine incision to delivery time, propofol vs. ketamine.*


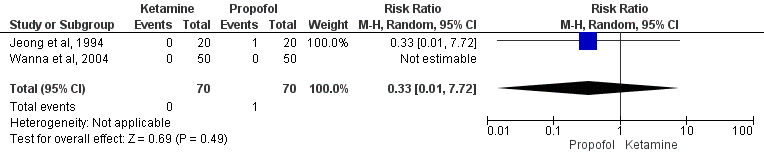


*Supplementary Figure 40: Comparison of number of newborns with Apgar score<7 at one minute, propofol vs. ketamine.*


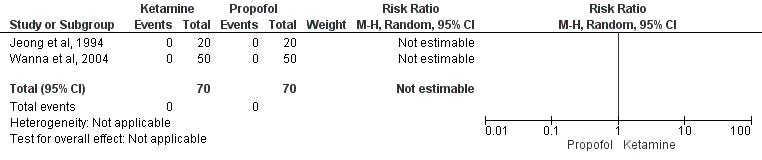


*Supplementary Figure 41: Comparison of the number of newborns with Apgar score<7 at five minutes, propofol vs. ketamine.*

*
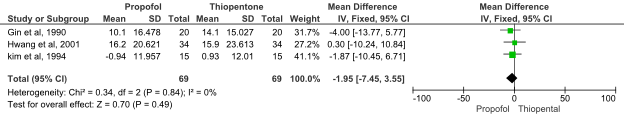
*

*Supplementary Figure 42: Change in SBP before and after induction of anesthesia, thiopentone vs. propofol.*

*
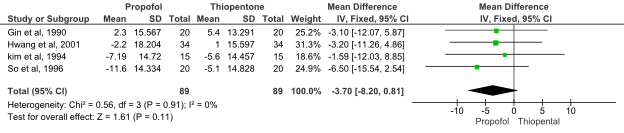
*

*Supplementary Figure 43: Change in DBP before and after induction of anesthesia, thiopentone vs. propofol.*

*
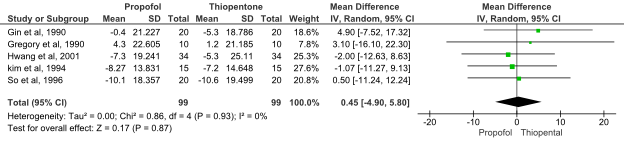
*

*Supplementary Figure 44: Change in HR before and after induction of anesthesia, thiopentone vs. propofol.*

*
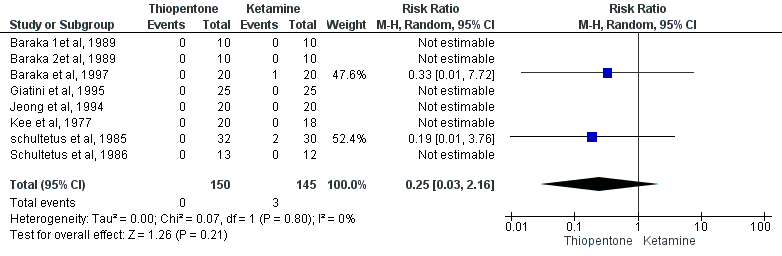
*

*Supplementary Figure 45: Leave one out meta-analysis of Apgar score<7 at 5 minutes after removal of Krissel et al, 1989, Thiopentone vs. Ketamine.*

*
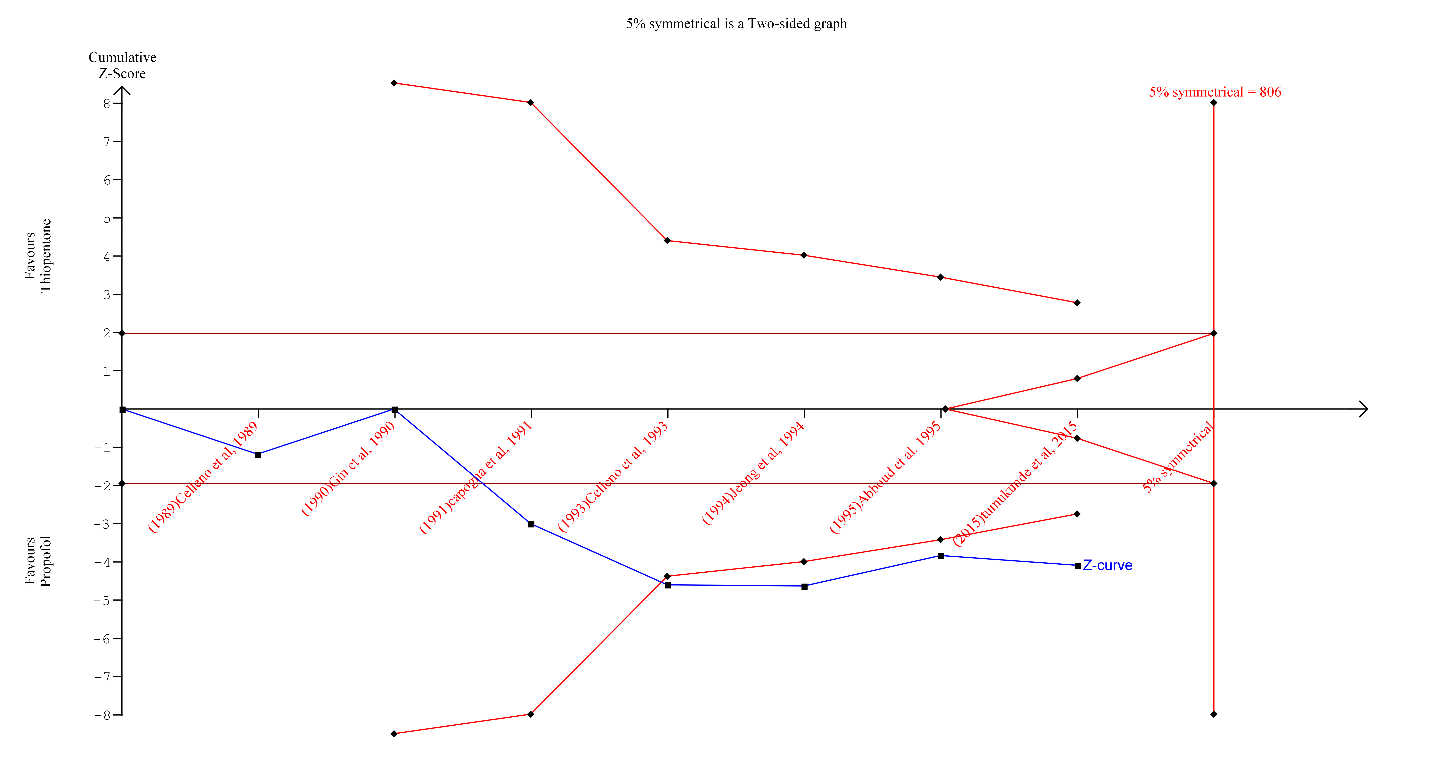
*

*Supplementary Figure 46: Trial sequential analysis (TSA) of the number of newborns with Apgar score < 7 at 1 min after delivery, thiopentone vs. propofol.*

*
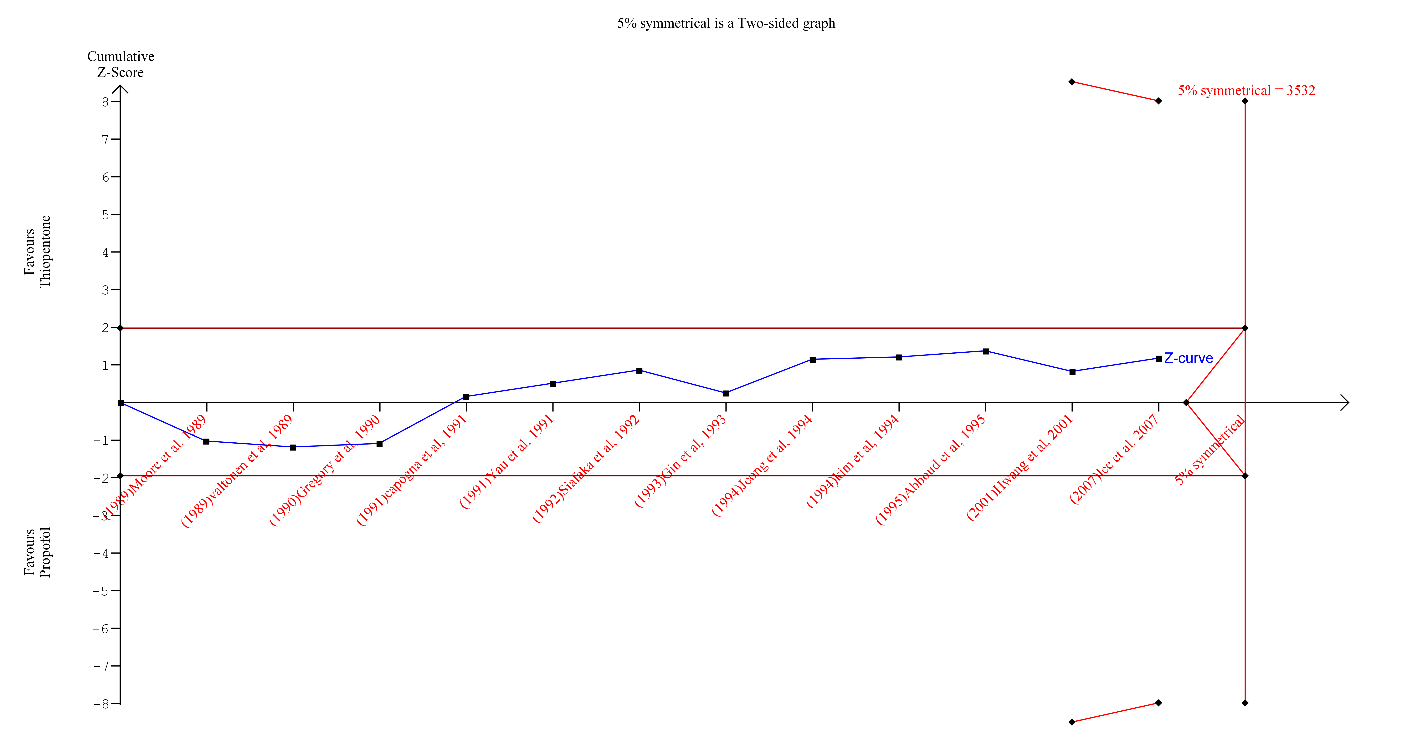
*

*Supplementary Figure 47: Trial sequential analysis (TSA) of the Pco2 in UA, thiopentone vs. Propofol.*
