# Supplementary material for: Hypnotics as induction agents for general anesthesia in cesarean section patients: updated systematic review and meta-analysis of randomized controlled trials
Source: J Anesth. 2025 Jun 21;39(6):948–75. doi: 10.1007/s00540-025-03524-8 (PMC12647321; doi:10.1007/s00540-025-03524-8)
Supplement: Supplementary file 2 — Supplementary file2 (DOCX 21 KB) [file 540_2025_3524_MOESM2_ESM.docx]

**Supplementary Table.** Details of the search strategy

| **Databases** | **Restrictions** | **Term** | **Number of items found** |
| --- | --- | --- | --- |
| PubMed | title and abstract | **1**= (“Cesarean Section*” OR “Abdominal Deliver*” OR “C-Section” OR “C Section” OR “C-Sections” OR “Caesarean Section*” OR “Postcesarean Section*” OR “surgical deliver*” OR “Surgical birth” OR “cesarean deliver*”)  **2**= (propofol OR Disoprofol OR “2,6-Bis(1-methylethyl)phenol” OR “2,6-Diisopropylphenol” OR “2,6 Diisopropylphenol” OR Diprivan OR Disoprivan OR “ICI-35,868” OR “ICI 35,868” OR “ICI35,868” OR “ICI-35868” OR “ICI35868” OR “ICI 35868” OR Aquafol OR Fresofol OR Ivofol OR Recofol OR Ciprofol OR Sediprofen OR Propoven OR “Propofol-Lipuro” OR Propofolum)  **3**= (thiopental OR Thiomebumal OR Thiopentobarbital OR Thiopentone OR Penthiobarbital OR Trapanal OR Nesdonal OR Pentothal OR “Pentothal Sodico” OR “Thiopental Sodium” OR Thionembutal OR “Thiopental Nycomed” OR Sodipental OR “Tiobarbital Braun” OR Bomathal OR Thiopentobarbitone OR “IntraPentothal OR 2-Thio-5-ethyl-5-sec-pentylbarbituric acid” OR “5-Ethyl-5-(1-methyl-butyl)-2-thioxo-dihydro-pyrimidine-4,6-dione” OR “Thiopentobarbituric acid” OR “Tiopentale”)  **4**= (ketamine OR ketimine OR Ketamina OR Ketaminum OR “2-(2-Chlorophenyl)-2-(methylamino)cyclohexanone” OR Ketalar OR “CI-581” OR “CI581” OR “CI 581” OR Ketanest OR “Ketamine Hydrochloride” OR Calipsol OR Calypsol OR Kalipsol OR Ketaset OR “2-(methylamino)-2-(2-chlorophenyl)cyclohexanone” OR “2-(o-chlorophenyl)-2-(methylamino)-cyclohexanone”)  **5**= (etomidate OR Etomidatum OR Ethomidate OR Amidate OR Hypnomidate OR R-26490 OR R26490 OR R 26490 OR Radenarkon OR “2-(1-Ethylphenyl)-1H-imidazole-5-carboxylate” OR “Ethyl 1-(1-phenylethyl)-1H-imidazole-5-carboxylate” OR Etomidato OR Etomidatum)  **6**= **2** OR **3** OR **4** OR **5**  **7**= **1** AND 6 | 646 |
| Scopus | title and abstract and keyword |  | 2796 |
| WOS | topic |  | 783 |
| Cochrane | title and abstract and keywords |  | 655 |
| Total | |  | 4880 |
|  | |  |  |

**PubMed**: Public/Publisher MEDLINE database; **WOS**: Web of Science; **Cochrane**: Cochrane Central Register of Controlled Trials; **Scopus**: Elsevier's abstract and citation database; **CI**: Compound Identifier; **R**: Research code or compound identifier ; *: Truncation symbol used in searches to retrieve variations of a root word.
