# Supplementary material for: Hypnotics as induction agents for general anesthesia in cesarean section patients: updated systematic review and meta-analysis of randomized controlled trials
Source: J Anesth. 2025 Jun 21;39(6):948–75. doi: 10.1007/s00540-025-03524-8 (PMC12647321; doi:10.1007/s00540-025-03524-8)
Supplement: Supplementary file 3 — Supplementary file3 (PDF 150 KB) [file 540_2025_3524_MOESM3_ESM.pdf]

Question: Thiopentone and Propofol compared to placebo in Cesarean Section

Bibliography: . Effect of hypnotics in Cesarean Section.

Certainty assessment							№ of patients		Effect		Certainty	Importance
№ of studies	Study design	Risk of bias	Inconsistency	Indirectness	Imprecision	Other considerations	Thiopentone and Propofol	placebo	Relative (95% CI)	Absolute (95% CI)		
**Apgar score<7 at 1 min after delivery**												
7	randomised trials	serious[a]	serious[b]	not serious	serious[c,d]	none	81/230 (35.2%)	43/230 (18.7%)	**RR 2.06** (1.06 to 4.01)	**198 more per 1,000** (from 11 more to 563 more)	⨁◯◯◯ Very low[a,b,c,d]	
**Apgar score<7 at 5 min after delivery**												
7	randomised trials	serious[a]	not serious	not serious	serious[c,d,e]	none	15/230 (6.5%)	8/230 (3.5%)	**RR 1.75** (0.81 to 3.78)	**26 more per 1,000** (from 7 fewer to 97 more)	⨁⨁◯◯ Low[a,c,d,e]	
**PH in UA**												
12	randomised trials	serious[a]	not serious	not serious	not serious[f]	none	263	266	-	MD **0.01 lower** (0.01 lower to 0)	⨁⨁⨁◯ Moderate[a,f]	
**BE in UA**												
8	randomised trials	serious[a]	serious[b]	not serious	serious[e]	none	177	175	-	MD **0.55 higher** (0.08 lower to 1.17 higher)	⨁◯◯◯ Very low[a,b,e]	
**SpO2 in UV**												
5	randomised trials	serious[a]	serious[b]	not serious	serious[c,d,e]	none	149	152	-	MD **0.5 lower** (6.7 lower to 5.7 higher)	⨁◯◯◯ Very low[a,b,c,d,e]	
**HCO3 in UA**												
3	randomised trials	serious[a]	serious[b]	not serious	serious[c,d,e]	none	78	80	-	MD **1.25 lower** (3.44 lower to 0.93 higher)	⨁◯◯◯ Very low[a,b,c,d,e]	
**Recovery duration**												
4	randomised trials	serious[a]	not serious	not serious	not serious[f]	none	91	91	-	MD **112.36 lower** (153.12 lower to 71.61 lower)	⨁⨁⨁◯ Moderate[a,f]	

CI: confidence interval; **MD:** mean difference; **RR:** risk ratio

Explanations

a. Downgraded due to risk of bias in studies (most studies have some concerns, and three studies of high risk)

b. Downgraded due to inconsistency (High heterogeneity)

c. Downgraded due to imprecision (Small sample size)

d. Downgraded due to imprecision (Wide confidence interval)

e. Downgraded due to imprecision (Confidence interval includes both benefit and harm.)

f. The confidence interval is narrow and supports a clear direction of effect

Question: Thiopentone and Ketamine compared to placebo in Cesarean Section

Bibliography: . Effect of hypnotics in Cesarean Section.

Certainty assessment							№ of patients		Effect		Certainty	Importance
№ of studies	Study design	Risk of bias	Inconsistency	Indirectness	Imprecision	Other considerations	Thiopentone and Ketamine	placebo	Relative (95% CI)	Absolute (95% CI)		
**pO2 in UA**												
6	randomised trials	serious[a]	not serious	not serious	serious[b,c]	none	135	141	-	MD **0.01 higher** (0.1 lower to 0.13 higher)	⨁⨁◯◯ Low[a,b,c]	
**pO2 in UV**												
9	randomised trials	serious[a]	not serious	not serious	not serious[d]	none	174	179	-	MD **0.24 higher** (0 to 0.47 higher)	⨁⨁⨁◯ Moderate[a,d]	
**Apgar score<7 at 1 min after delivery**												
9	randomised trials	serious[a]	not serious	not serious	not serious[d]	none	18/175 (10.3%)	35/170 (20.6%)	**RR 0.54** (0.32 to 0.90)	**95 fewer per 1,000** (from 140 fewer to 21 fewer)	⨁⨁⨁◯ Moderate[a,d]	
**Apgar score<7 at 5 min after delivery**												
9	randomised trials	serious[a]	not serious	not serious	not serious[d]	none	3/175 (1.7%)	13/170 (7.6%)	**RR 0.28** (0.10 to 0.79)	**55 fewer per 1,000** (from 69 fewer to 16 fewer)	⨁⨁⨁◯ Moderate[a,d]	
**Accidental awareness under General Anesthesia (AAGA)**												
4	randomised trials	serious[a]	not serious	not serious	not serious[d]	none	31/70 (44.3%)	9/70 (12.9%)	**RR 3.18** (1.75 to 5.77)	**280 more per 1,000** (from 96 more to 613 more)	⨁⨁⨁◯ Moderate[a,d]	
**PH in UA**												
4	randomised trials	serious[a]	not serious	not serious	serious[b,c]	none	65	66	-	MD **0** (0.01 lower to 0.01 higher)	⨁⨁◯◯ Low[a,b,c]	
**PH in UV**												
5	randomised trials	serious[a]	not serious	not serious	serious[b,c]	none	85	86	-	MD **0** (0.02 lower to 0.02 higher)	⨁⨁◯◯ Low[a,b,c]	

CI: confidence interval; **MD:** mean difference; **RR:** risk ratio

Explanations

a. Downgraded due to risk of bias in studies (most studies have some concerns, and three studies of high risk)

b. Downgraded due to imprecision (Small sample size)

c. Downgraded due to imprecision (Confidence interval includes both benefit and harm)

d. The confidence interval is narrow and supports a clear direction of effect

Question: Propofol and Ketamine compared to placebo in Cesarean Section

Bibliography: . Effect of hypnotics in Cesarean Section.

Certainty assessment							№ of patients		Effect		Certainty	Importance
№ of studies	Study design	Risk of bias	Inconsistency	Indirectness	Imprecision	Other considerations	Propofol and Ketamine	placebo	Relative (95% CI)	Absolute (95% CI)		
**pO2 in UA**												
2	randomised trials	serious[a]	serious[b]	not serious	serious[c,d]	none	35	35	-	MD **0.09 higher** (0.05 lower to 0.23 higher)	⊕◯◯◯ Very low[a,b,c,d]	
**pCO2 in UV**												
2	randomised trials	serious[a]	serious[b]	not serious	serious[c,d]	none	35	35	-	MD **0.04 higher** (0.57 lower to 0.65 higher)	⊕◯◯◯ Very low[a,b,c,d]	
**Apgar score<7 at 1 min after delivery**												
2	randomised trials	serious[a]	serious[b]	not serious	serious[c,d,e]	none	0/70 (0.0%)	1/70 (1.4%)	**RR 0.33** (0.01 to 7.72)	**10 fewer per 1,000** (from 14 fewer to 96 more)	⊕◯◯◯ Very low[a,b,c,d,e]	
**Apgar score<7 at 5 min after delivery**												
2	randomised trials	serious[a]	serious[b]	not serious	serious[c,d,e]	none	0/70 (0.0%)	0/70 (0.0%)	not pooled	see comment	⊕◯◯◯ Very low[a,b,c,d,e]	
**PH in UA**												
2	randomised trials	serious[a]	serious[b]	not serious	serious[c,d]	none	35	35	-	MD **0** (0.01 lower to 0.01 higher)	⊕◯◯◯ Very low[a,b,c,d]	
**PH in UV**												
2	randomised trials	serious[a]	serious[b]	not serious	serious[c,d]	none	35	35	-	MD **0.01 lower** (0.03 lower to 0.02 higher)	⊕◯◯◯ Very low[a,b,c,d]	

CI: confidence interval; **MD:** mean difference; **RR:** risk ratio

Explanations

a. Downgraded due to risk of bias in studies (studies have some concerns)

b. Downgraded due to inconsistency (High heterogeneity)

c. Downgraded due to imprecision (Small sample size)

d. Downgraded due to imprecision (Confidence interval includes both benefit and harm)

e. Downgraded due to imprecision (Wide confidence interval)
